# Supplementary material for: PD-1 Controls Follicular T Helper Cell Positioning and Function
Source: Immunity. 2018 Aug 21;49(2):264–274.e4. doi: 10.1016/j.immuni.2018.06.012 (PMC6104813; doi:10.1016/j.immuni.2018.06.012)
Supplement: Document S1. Figures S1–S7 [file mmc1.pdf]

Immunity, Volume *49*

Supplemental Information

PD-1 Controls Follicular T Helper Cell Positioning and Function

Jingwen Shi, Shiyue Hou, Qian Fang, Xin Liu, Xiaolong Liu, and Hai Qi

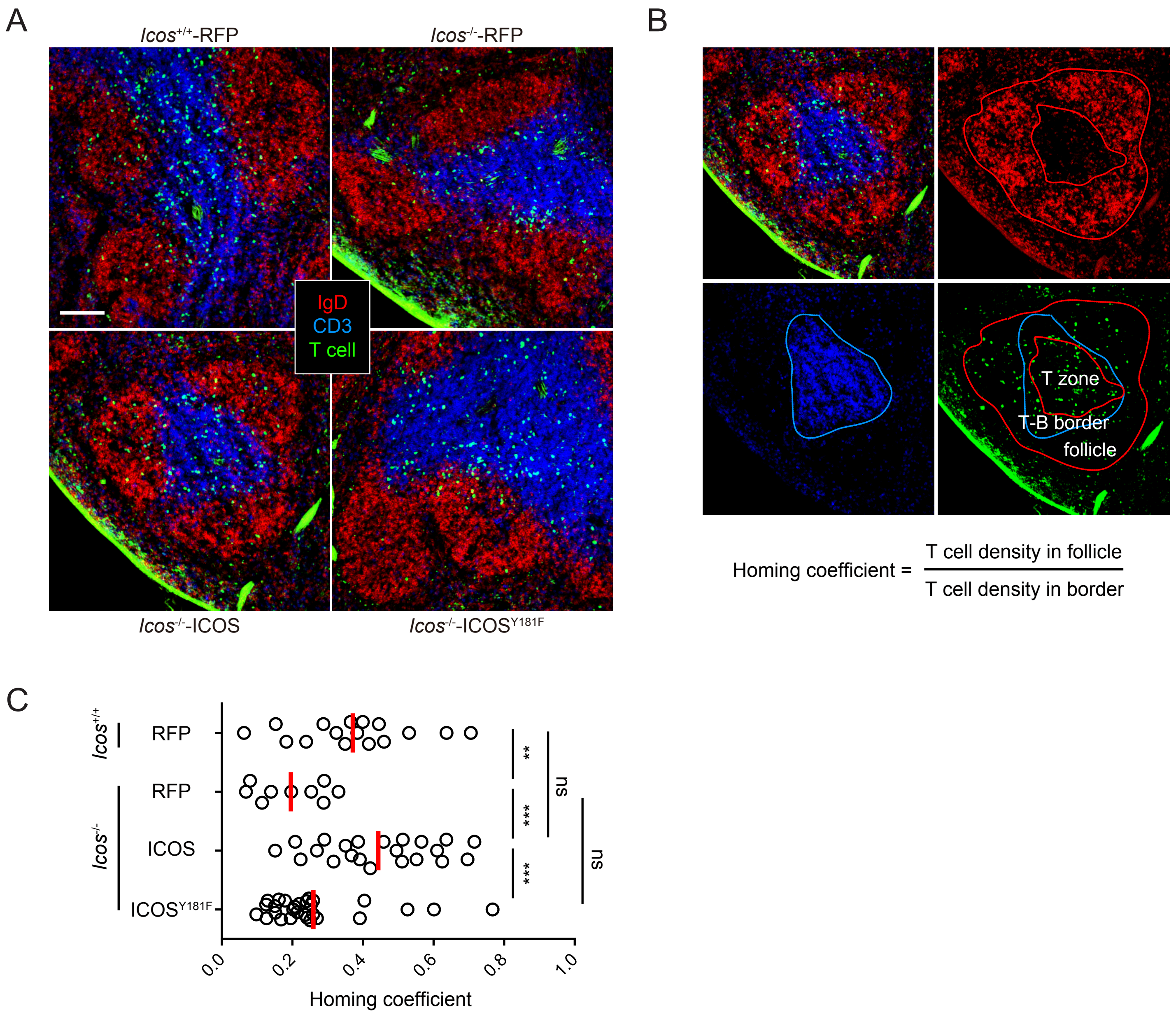

Figure S1. ICOS-driven follicular recruitment requires its PI3K-activating Y181-based motif. Related to Figure 1.

T cells of indicated genotypes were retrovirally co-transduced with a vector co-expressing CXCR5 and GFP and another vector expressing empty RFP, wildtype ICOS or mutant ICOS^{Y181F}. These cells were transferred into B6 mice (2-3×10^{6} sorted GFP^{+}RFP^{+} cells per mouse).

(A) GFP^{+} T-cell distribution patterns in the spleen 24h after transfer. Scale bar, 100 µm.

(B) Derivation of the follicular homing coefficient parameter. The outer boundary of the T-cell zone is defined as endogenous CD3^{+} cells farthest into the follicle (blue line), while the inner boundary of the follicle is defined as endogenous IgD^{+} cells farthest into the T-cell zone (inner red line). The T–B border area is thus the region in-between endogenous CD3^{+} cells farthest into the follicle and endogenous IgD^{+} cells farthest into the T-cell zone.

(C) Homing coefficients of the four groups in (A). Each symbol represents one section. Data represent four independent experiments, each involving at least 3 recipients.

** P<0.01; *** P<0.0001; ns, not significant.

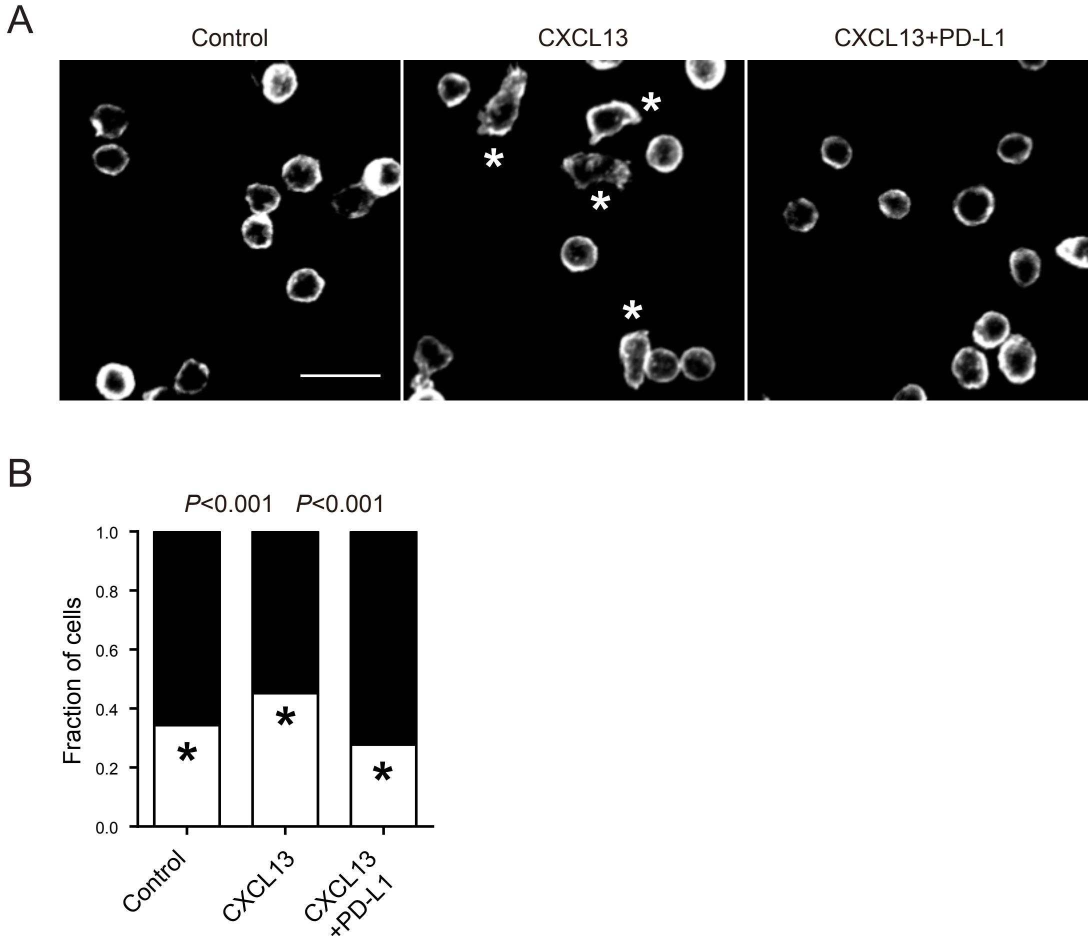

Figure S2. PD-1 inhibits CXCL13-stimulated T-cell polarization. Related to Figure 1.

(A) Typical morphologies revealed by Phalloidin staining of F-actin in co-transduced CXCR5^{+}PD-1^{+} T cells that were not stimulated (Control), CXCL13-stimulated (CXCL13), or CXCL13-stimulated in the presence of PD-L1-Fc (CXCL13+PD-L1). Asterisks mark polarized cells. Scale bar, 20 μm.

(B) Fractions of polarized (*) and non-polarized cells among the 3 groups, with a total of 1629 (Control), 1375 (CXCL13), and 1815 (CXCL13+PD-L1) cells enumerated in 4 independent experiments. *P* values from Fisher's exact tests.

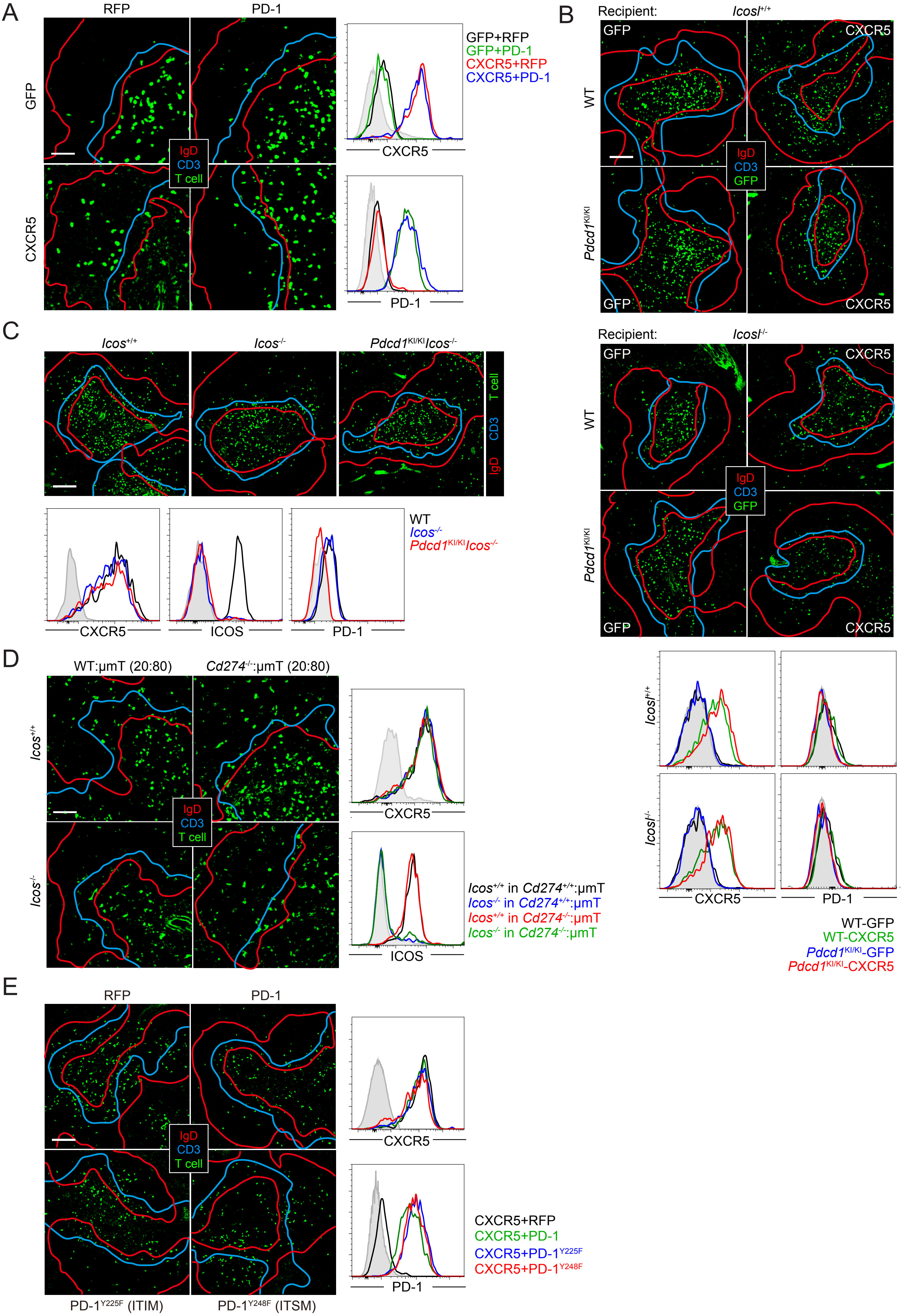

A
RFP
PD-1
GFP
CXCR5
IgD
CD3
T cell
GFP+RFP
GFP+PD-1
CXCR5+RFP
CXCR5+PD-1
CXCR5
PD-1
B
Recipient:
Icosl+/+
WT
Pdcd1KI/KI
GFP
CXCR5
IgD
CD3
GFP
Recipient:
Icosl-/-
WT-GFP
WT-CXCR5
Pdcd1KI/KI-GFP
Pdcd1KI/KI-CXCR5
Icosl+/+
Icosl-/-
CXCR5
PD-1
C
Icos+/+
Icos-/-
Pdcd1KI/KIIcos-/-
T cell
CD3
IgD
WT
Icos-/-
Pdcd1KI/KIIcos-/-
CXCR5
ICOS
PD-1
D
WT:μmT (20:80)
Cd274-/-:μmT (20:80)
Icos+/+
Icos-/-
IgD
CD3
T cell
CXCR5
ICOS
Icos+/+ in Cd274+/+:μmT
Icos-/- in Cd274+/+:μmT
Icos+/+ in Cd274-/-:μmT
Icos-/- in Cd274-/-:μmT
E
RFP
PD-1
IgD
CD3
T cell
PD-1Y225F (ITIM)
PD-1Y248F (ITSM)
CXCR5
PD-1
CXCR5+RFP
CXCR5+PD-1
CXCR5+PD-1Y225F
CXCR5+PD-1Y248F

Figure S3. Definition of T-zone and follicular borders for quantitative homing analyses and surface levels of CXCR5, PD-1 and ICOS on transduced cells. Related to Figure 1.
The boundaries of T-cell zones, follicles and T-B border areas are defined as in Figure S1B. Outlines of T-cell zones, follicles, and T-B border areas for representative images from all the homing assays shown in Figures 1D (**A**), 2A (**B**), 2C (**C**), 3A (**D**) and 4A (**E**), respectively. Surface expression of CXCR5 and PD-1, together with ICOS in certain experiments, are also shown for the transferred cells in corresponding experiments.

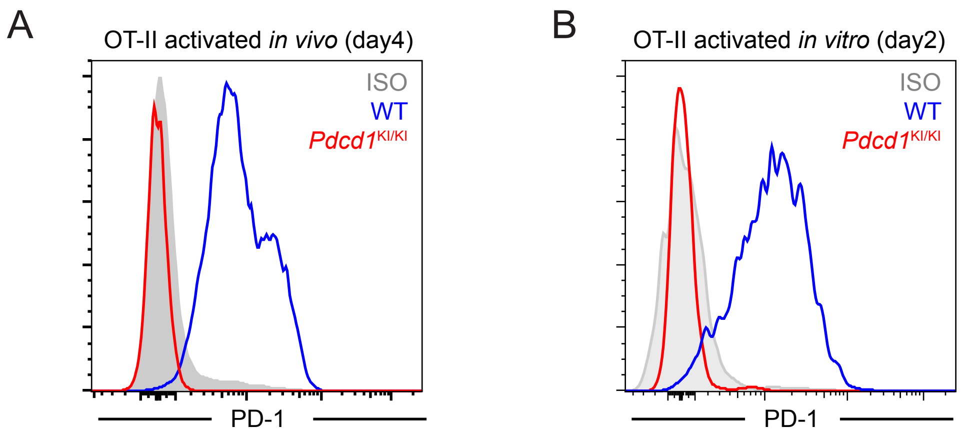

Figure S4. Disabled PD-1 upregulation in *Pdcd1*$^{KI/KI}$ T cells. Related to Figure 2.
Surface PD-1 expression by wildtype or *Pdcd1*$^{KI/KI}$ OT-II T cells in adoptive B6 hosts 4 d after ovalbulmin immunization (A), or 2 d after in vitro activation with plate-bounded anti-CD3 and anti-CD28 (B). Grey histograms, isotype control. Data represent three independent experiments.

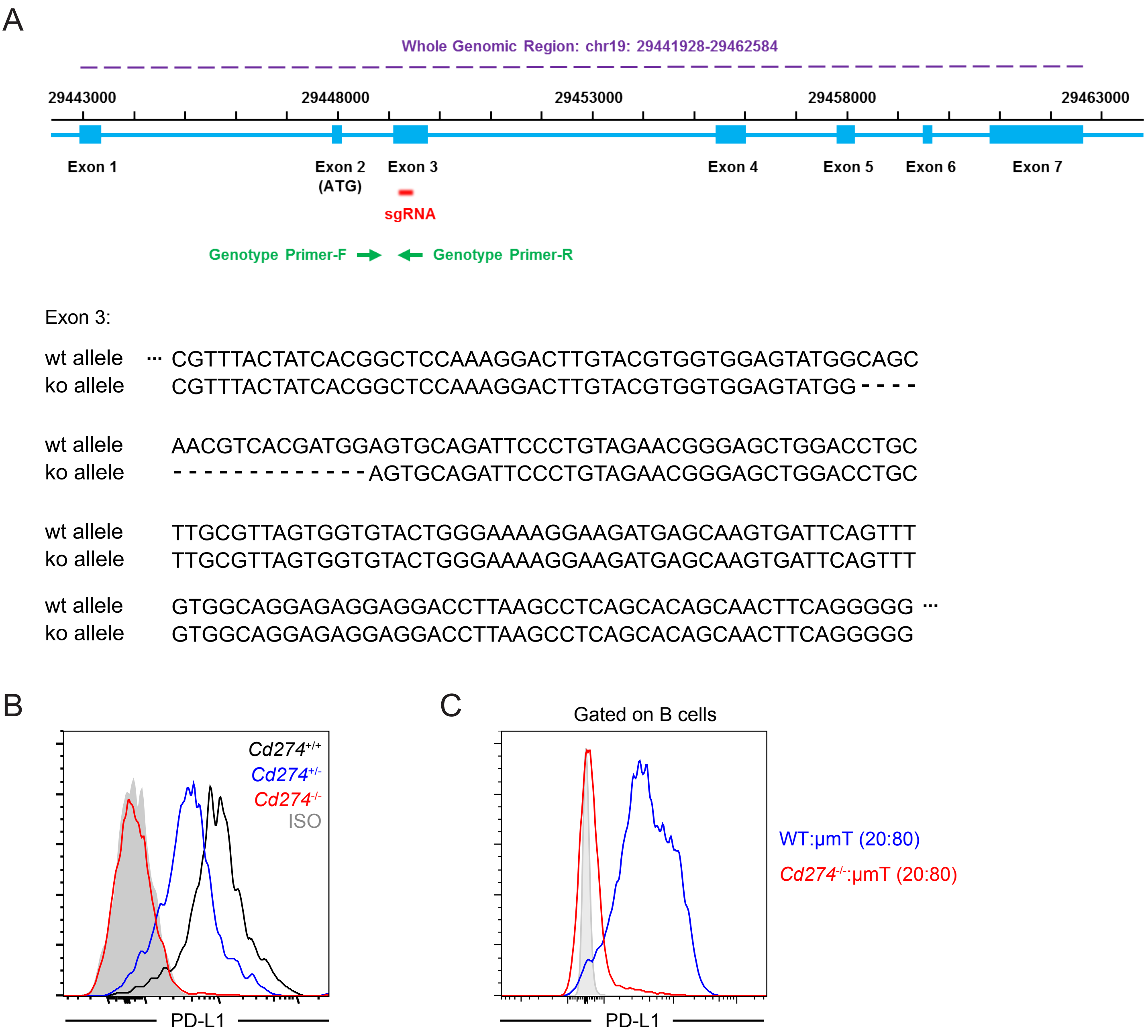

Figure S5. Genetic ablation of *Cd274*. Related to Figure 3.

(A) Schematic presentation of CRISPR/Cas9-mediated targeting and genotyping strategy for genomic deletion of *Cd274* (top) and sequence alignment of the wildtype allele and the knockout allele used in this study (bottom). Nucleotide coordinates according to NCBI and exon distribution of the *Cd274* locus are shown. The exon 3 was targeted by an sgRNA (red), and 2 green arrows mark the positions of the genotyping primers (see Methods for primer sequences).

(B) PD-L1 expression by naïve B cells from mice of the indicated genotypes.

(C) PD-L1 expression by B cells from mixed BM chimeras of indicated types, as used in Figure 3. Grey histogram, isotype control.

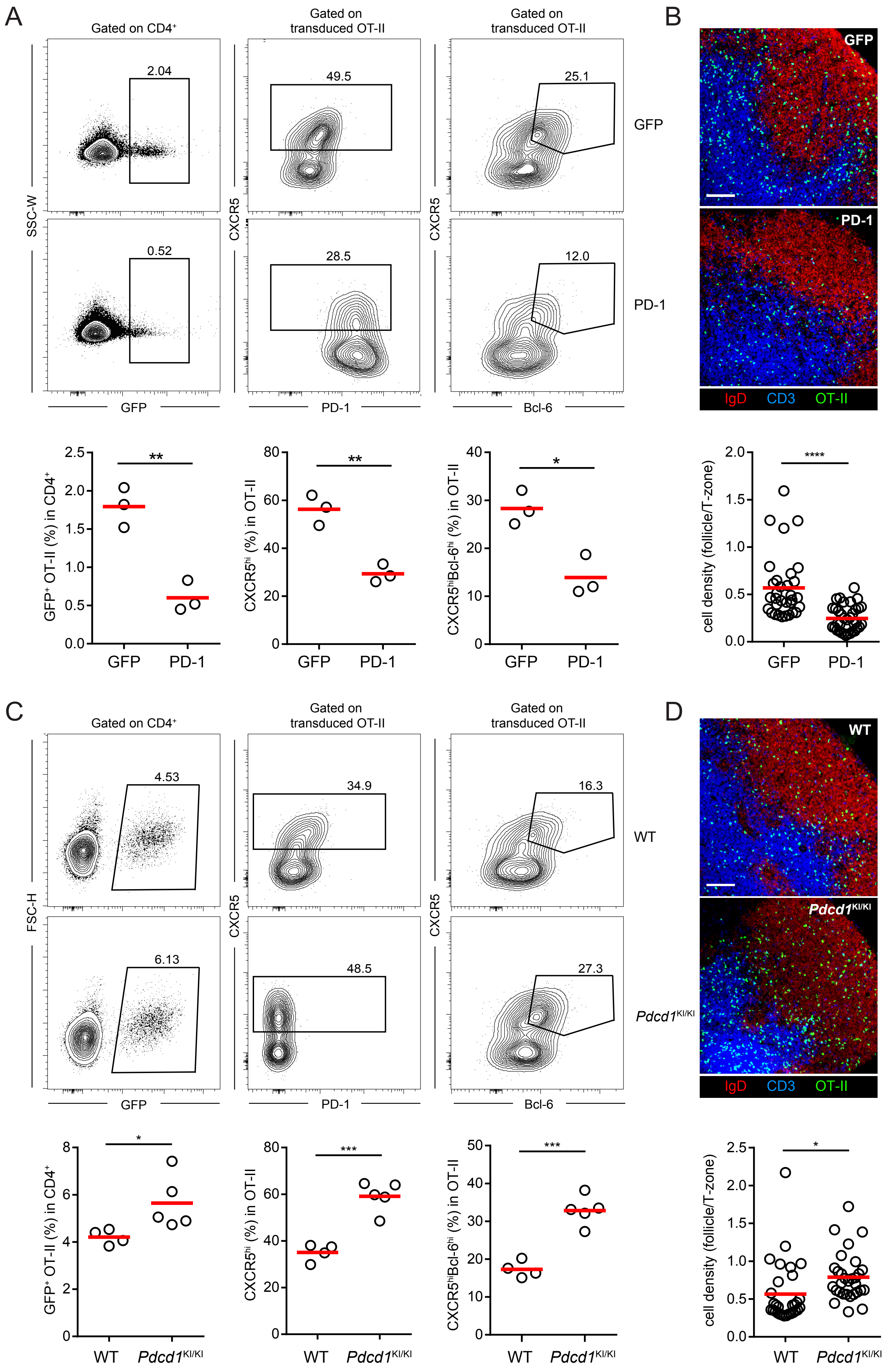

A
Gated on CD4+
Gated on transduced OT-II
Gated on transduced OT-II
2.04
49.5
25.1
GFP
0.52
28.5
12.0
PD-1
SSC-W
CXCR5
GFP
PD-1
Bcl-6
B
GFP
PD-1
IgD CD3 OT-II
GFP+ OT-II (%) in CD4+
CXCR5hi (%) in OT-II
CXCR5hiBcl-6hi (%) in OT-II
cell density (follicle/T-zone)
**
**
*
****
GFP PD-1
C
Gated on CD4+
Gated on transduced OT-II
Gated on transduced OT-II
4.53
34.9
16.3
WT
6.13
48.5
27.3
Pdcd1KI/KI
FSC-H
CXCR5
GFP
PD-1
Bcl-6
D
WT
Pdcd1KI/KI
IgD CD3 OT-II
*
***
***
*
WT Pdcd1KI/KI

Figure S6. PD-1 inhibits Tfh development. Related to Figure 5.

(A) OT-II T cells transduced with a vector expressing PD-1 or control GFP were transferred into B6 mice (5×10^5 sorted GFP^+ cells per mouse), and then these mice were subcutaneously immunized with OVA protein. Expansion of GFP^+ OT-II cells, measured by their frequencies in total CD4 T cells, and Tfh frequencies, as measured by $CXCR5^{hi}$ or $CXCR5^{hi}Bcl\text{-}6^{hi}$ fractions in OT-II cells were assayed in the draining lymph node 4 d post immunization.

(B) OT-II distribution patterns and density ratios between the follicle and the T-zone in draining lymph nodes in (A).

(C) GFP-transduced wildtype or *Pdcd1*$^{KI/KI}$ OT-II T cells were transferred into B6 mice (5×10^5 sorted GFP^+ cells per mouse), and the recipients were subcutaneously immunized with OVA protein. Expansion of GFP^+ OT-II cells, measured by their frequencies in total CD4 T cells, and Tfh frequencies, as measured by $CXCR5^{hi}$ or $CXCR5^{hi}Bcl\text{-}6^{hi}$ fractions in OT-II cells were assayed in the draining lymph node 4 d post immunization.

(D) OT-II distribution patterns and density ratios between the follicle and the T-zone in draining lymph nodes in (C).

Scale bar, 100 μm. Flow cytometry data are from one of 3 independent experiments with similar results (3-4 recipient mice per condition per experiment). Data on cell density ratios are pooled from 3 independent experiments. * $P<0.05$; ** $P<0.01$; **** $P<0.0001$.

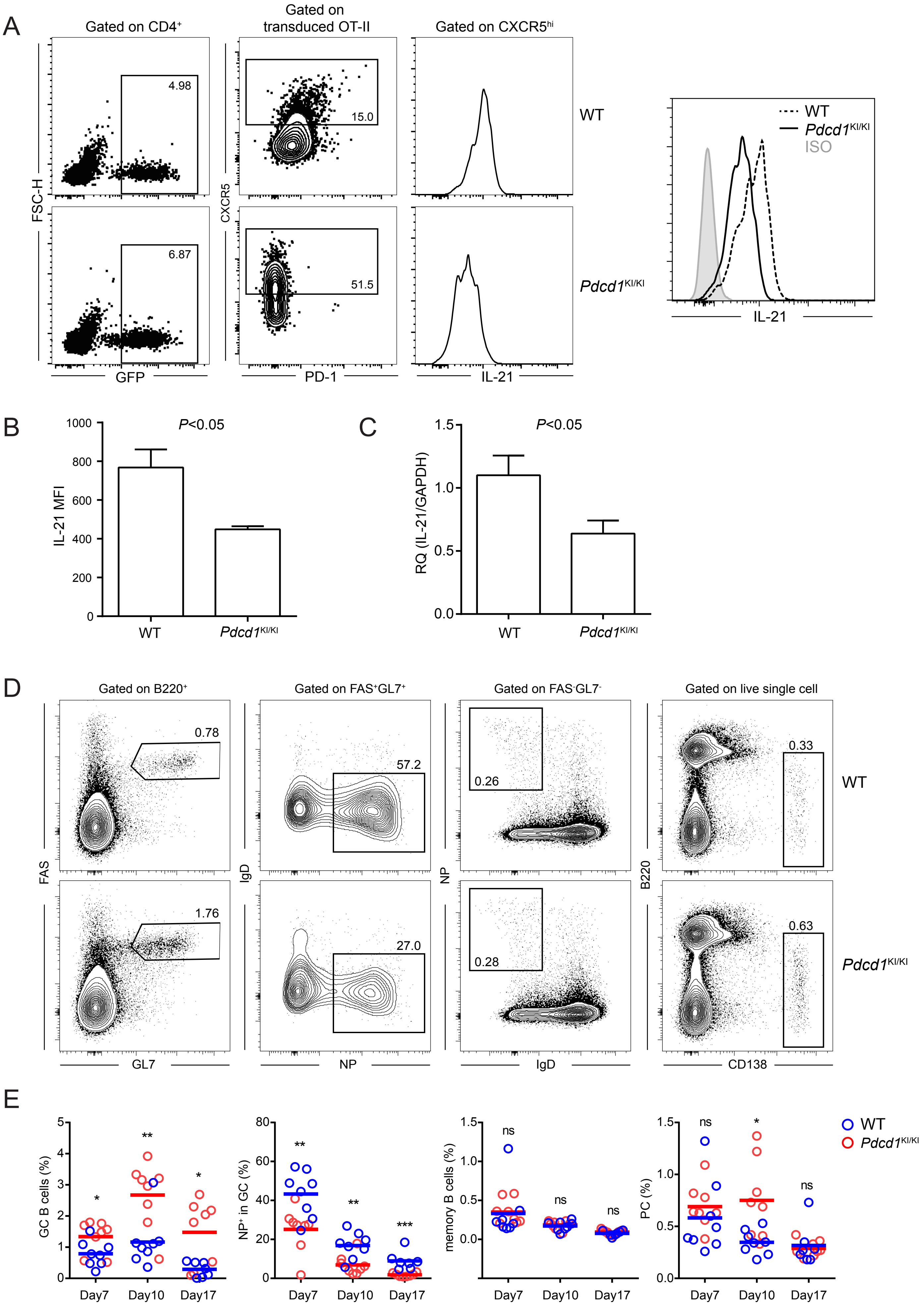

A
Gated on CD4+
Gated on transduced OT-II
Gated on CXCR5hi
4.98
15.0
6.87
51.5
WT
Pdcd1KI/KI
FSC-H
CXCR5
GFP
PD-1
IL-21
WT
Pdcd1KI/KI
ISO
IL-21
B
IL-21 MFI
P<0.05
WT
Pdcd1KI/KI
C
RQ (IL-21/GAPDH)
P<0.05
WT
Pdcd1KI/KI
D
Gated on B220+
Gated on FAS+GL7+
Gated on FAS-GL7-
Gated on live single cell
0.78
57.2
0.26
0.33
1.76
27.0
0.28
0.63
WT
Pdcd1KI/KI
FAS
IgD
NP
B220
GL7
NP
IgD
CD138
E
GC B cells (%)
NP+ in GC (%)
memory B cells (%)
PC (%)
Day7
Day10
Day17
ns
WT
Pdcd1KI/KI

Figure S7. *Pdcd1*$^{KI/KI}$ Tfh cells are impaired in IL-21 production and causes abnormal GC responses. Related to Figure 7.

(A) GFP-transduced wildtype or *Pdcd1*$^{KI/KI}$ OT-II T cells were transferred into B6 mice (5×10^5 sorted GFP$^+$ cells per mouse) together with 5×10^5 dsRed-expressing MD4 B cells, and the recipients were subcutaneously immunized with HEL-OVA. Strategies for gating GFP$^+$CXCR5^{hi} OT-II Tfh cells and intracellular IL-21 staining of Tfh cells of the two genotypes are shown.

(B) Mean fluorescence intensity (MFI, mean±sem) of IL-21 staining in Tfh cells of indicated genotypes (n=3). One of two experiments with similar results is shown.

(C) Relative IL-21 expression in wildtype or *Pdcd1*$^{KI/KI}$ OT-II Tfh cells as detected by quantitative RT-PCR. Data are mean ± s.e.m of 5 independent experiments.

(D) *Sap*$^{-/-}$ mice received 5×10^5 wildtype or *Pdcd1*$^{KI/KI}$ OT-II T cells and were intraperitoneally immunized with NP-OVA. From left to right, gating of GC B cells, NP-binding cells in GCs, memory B cells, and plasma cells, as assayed 7 d post immunization.

(E) Frequencies of GC B cells in total B220$^+$ cells, NP-specific cells in total GC B cells, NP-specific memory B cells in non-GC B cells, and plasma cells in total splenocytes 7, 10, and 17 d after immunization. Each symbol denotes one mouse. Data are pooled from 3 independent experiments.

* P<0.05, ** P<0.01, *** P<0.001.
