# Supplementary material for: PD-1 Controls Follicular T Helper Cell Positioning and Function
Source: Immunity. 2018 Aug 21;49(2):264–274.e4. doi: 10.1016/j.immuni.2018.06.012 (PMC6104813; doi:10.1016/j.immuni.2018.06.012)
Supplement: Document S2. Article plus Supplemental Information [file mmc3.pdf]

Immunity

Article

PD-1 Controls Follicular T Helper Cell Positioning and Function

Graphical Abstract

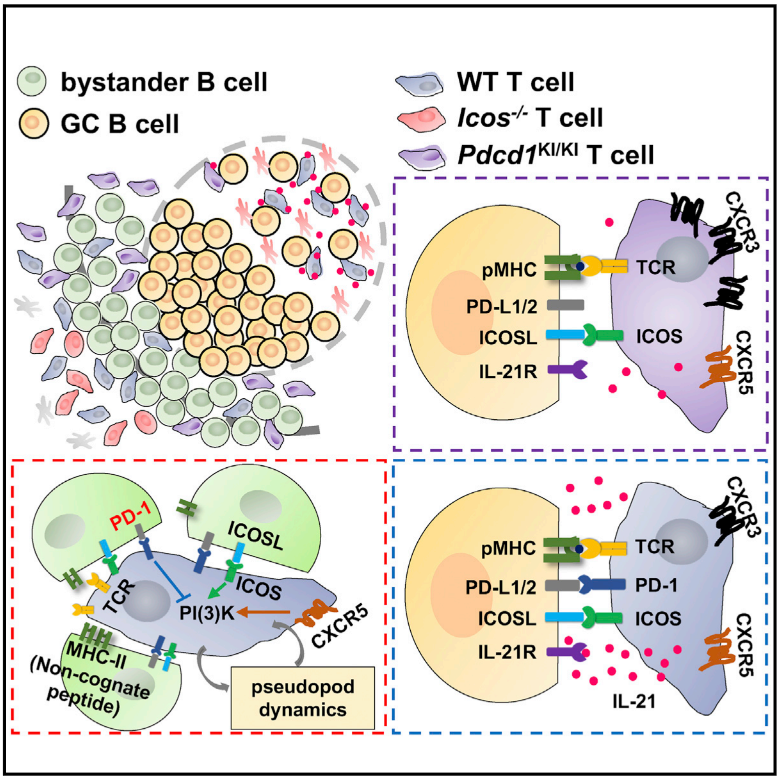

Highlights

- PD-1 suppresses follicular T cell recruitment
- Bystander B cells regulate follicular T cell recruitment by both ICOSL and PD-L1
- PD-1 limits CXCR3 expression to confine Tfh cell localization in the GC
- PD-1-PD-L1 interactions increase the stringency of GC affinity selection

Authors

Jingwen Shi, Shiyue Hou, Qian Fang, Xin Liu, Xiaolong Liu, Hai Qi

Correspondence

qihai@tsinghua.edu.cn

In Brief

Tfh cells highly express the inhibitory PD-1 molecule, although its function in the germinal center response is not well understood. Shi et al. show that, by operating in both costimulatory and bystander signaling mode, PD-1 suppresses follicular T cell recruitment but promotes Tfh cell concentration in the GC territory and helps maintain the stringency of GC affinity selection.

Shi et al., 2018, Immunity *49*, 264–274
August 21, 2018 © 2018 The Authors. Published by Elsevier Inc.
https://doi.org/10.1016/j.immuni.2018.06.012

CellPress

Immunity
Article

PD-1 Controls Follicular T Helper Cell Positioning and Function

Jingwen Shi,[1,2,3,4,5] Shiyue Hou,[1,2,3,5] Qian Fang,[1,2,3,4,5] Xin Liu,[1,2,3,5] Xiaolong Liu,[6] and Hai Qi[1,2,3,4,5,7,*]
[1]Tsinghua-Peking Center for Life Sciences, Tsinghua University, Beijing 100084, China
[2]Laboratory of Dynamic Immunobiology, Institute for Immunology, Tsinghua University, Beijing 100084, China
[3]Department of Basic Medical Sciences, School of Medicine, Tsinghua University, Beijing 100084, China
[4]School of Life Sciences, Tsinghua University, Beijing 100084, China
[5]Beijing Key Lab for Immunological Research on Chronic Diseases, Tsinghua University, Beijing 100084, China
[6]State Key Laboratory of Cell Biology, Chinese Academy of Sciences Center for Excellence in Molecular Cell Science, Institute of Biochemistry and Cell Biology, Shanghai Institutes for Biological Sciences, Chinese Academy of Sciences, Shanghai 200031, China
[7]Lead Contact
*Correspondence: qihai@tsinghua.edu.cn
https://doi.org/10.1016/j.immuni.2018.06.012

SUMMARY

Follicular T helper (Tfh) cells highly express the programmed cell death-1 (PD-1) molecule. Whereas inhibition of T cell receptor (TCR) signaling and CD28 co-stimulation is thought to be the primary mode of PD-1 functions, whether and how PD-1 regulates Tfh cell development and function is unclear. Here we showed that, when engaged by the ensemble of bystander B cells constitutively expressing PD-1 ligand 1 (PD-L1), PD-1 inhibited T cell recruitment into the follicle. This inhibition involved suppression of PI3K activities downstream of the follicle-guidance receptor CXCR5, was independent of co-signaling with the TCR, and necessitated ICOS signaling to overcome. PD-1 further restricted CXCR3 upregulation on Tfh cells, serving to concentrate these cells toward the germinal center territory, where PD-L1-PD-1 interactions between individual Tfh and B cells optimized B cell competition and affinity maturation. Therefore, operating in both costimulation-independent and -dependent manners, PD-1 controls tissue positioning and function of Tfh cells.

INTRODUCTION

Follicular T helper (Tfh) cells are the effector T cell subset that is specialized in promoting the T cell-dependent B cell response and the germinal center (GC) reaction (Crotty, 2011; Qi, 2016; Vinuesa et al., 2016). These cells express the BCL-6 transcription regulator (Johnston et al., 2009; Nurieva et al., 2009; Yu et al., 2009) and exhibit a unique CXCR5^{hi}CCR7^{lo}PD-1^{+}ICOS^{+} surface phenotype. To develop into Tfh cells, naive T cells are initially activated in the T cell zone by dendritic cells (Deenick et al., 2010; Goenka et al., 2011). Antigen activation not only causes upregulation of BCL-6, ICOS, and PD-1 but also leads to CXCR5 upregulation and CCR7 downregulation, which together guide activated T cells to relocate toward the T zone-follicle border (T-B border) and enter the follicle (Vinuesa and Cyster, 2011). At the T-B border and particularly inside the follicle, T cells not only escape from high concentrations of IL-2 and IL-7 that inhibit BCL-6 expression (Ballesteros-Tato et al., 2012; Johnston et al., 2012; McDonald et al., 2016; Nurieva et al., 2012) but also experience IL-6 produced locally that helps to promote Tfh cell development and maintain the Tfh identity (Harker et al., 2011; Nurieva et al., 2008; Qi, 2016).

Signaling through B7-family costimulatory molecules CD28 and ICOS is important for Tfh cell development. CD28 co-stimulates for T cell priming, whereas ICOS co-stimulation promotes the CXCR5 upregulation (Choi et al., 2011). Moreover, in a costimulation-independent manner that requires ICOSL expression by follicular bystander B cells, ICOS signaling optimizes phosphoinositide-3 kinase (PI3K)-dependent pseudopod dynamics, promotes T cell persistent motility at the T-B border, and thereby enables CXCR5-expressing T cells to not only sense the CXCL13 gradient emanating from the follicle but also efficiently migrate across the border and into the follicle (Xu et al., 2013). Constitutive ICOS signaling in the follicle is required for maintaining the follicular residence of T cells (Weber et al., 2015). ICOS is important not only for follicular recruitment and maintenance of Tfh cells but also for help-delivering functions of Tfh cells. Tfh cells interact with antigen-specific B cells and provide help signals such as CD40L through cell-cell contacts. ICOS co-stimulates calcium fluxes in Tfh cells during antigen-specific interactions with GC B cells, promotes T-B contacts in the form of entanglement, and facilitate rapid selection of high-affinity B cells through the ICOS-CD40 intercellular positive feedback between the two types of cells (Liu et al., 2015). Therefore, ICOS functions in both a costimulation-dependent and -independent manner to promote Tfh cell development and function. However, given that chemokine receptors signal to PI3K, and that CXCL13, which is amply available at the T-B border and in the follicle, could presumably activate PI3K in CXCR5-expressing T cells, it remains unresolved as to why ICOS is required for persistent T cell motility at the T-B border and in the follicle but not for migration in the T cell zone.

This is an open access article under the CC BY license (http://creativecommons.org/licenses/by/4.0/).

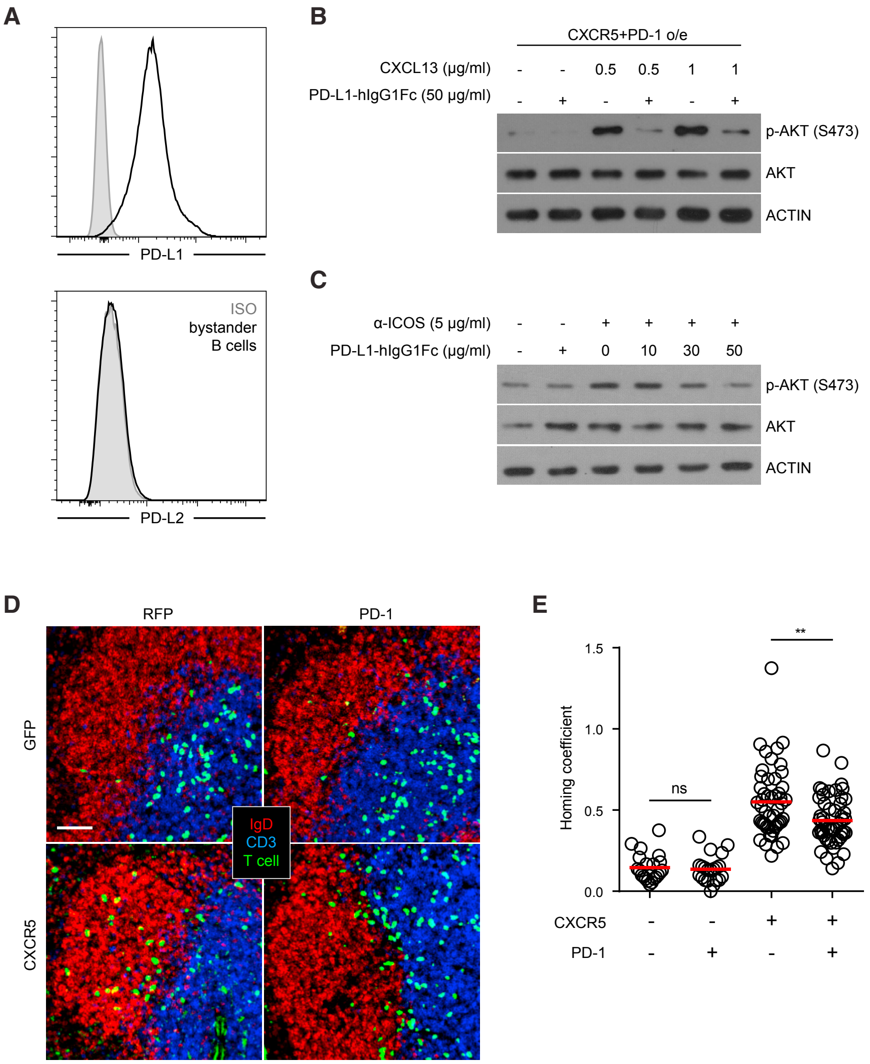

Figure 1. Costimulation-Independent Suppression of PI3K Activities and Follicular Recruitment of T Cells by PD-1

(A) Surface staining of PD-L1 or PD-L2 expression on follicular B cells. Grey histograms: isotype control staining.

(B) $CD4^+$ T cells retrovirally co-transduced with CXCR5 and PD-1 were starved in serum-free media for 3 hr. AKT phosphorylation was probed after 30 min CXCL13 stimulation at indicated concentrations in the presence or absence of PD-L1-Fc crosslinked by anti-human IgG. Data represent two independent experiments.

(C) AKT phosphorylation was probed after $CD4^+$ T cells were starved in serum-free media for 3 hr and then stimulated with anti-ICOS in the presence or absence of PD-L1-Fc at indicated concentration for 30 min. Data represent two independent experiments.

(D) Splenic distribution patterns of $CD4^+$ T cells retrovirally co-transduced with a combination of CXCR5 or control GFP and PD-1 or control RFP 24 hr after being injected into B6 mice (2–3 × 10^6 sorted transduced cells per mouse).

(E) Scatterplots of the homing coefficients of the four groups in (D), with each symbol indicating one section.

Data are pooled from four independent experiments, with each experiment contributing 10–20 sections. Scale bar, 50 μm. $^{**}p < 0.01$; ns, not significant.

PD-1 is another B7-family molecule that, albeit inhibitory (Keir et al., 2008), is highly expressed by Tfh cells, particularly those localized inside the GC territory (Haynes et al., 2007). For antigen-experienced $CD8^+$ T cells, high expression of PD-1 is associated with functional exhaustion (Barber et al., 2006; Wherry and Kurachi, 2015). This is because the cytoplasmic domain of PD-1 contains an immunotyrosine-based inhibitory motif (ITIM) and an immunotyrosine-based switch motif (ITSM) and can recruit tyrosine phosphatases to dephosphorylate signaling components downstream of TCR and CD28 pathways (Hui et al., 2017; Parry et al., 2005; Yokosuka et al., 2012). In contrast, Tfh cells are highly functional and sensitive to antigen presented by cognate B cells. It is thought that the heightened PD-1 expression on Tfh cells simply reflects experience of antigen stimulation, especially when inside the GC, and Tfh cells have to function despite the PD-1-mediated inhibition. Alternatively, inhibitory functions of PD-1 might play a positive role in Tfh cell development and function.

In our search for a mechanistic explanation for the requirement of ICOS to promote T cell migration at the T-B border but not in the T cell-zone, we found that, in a cosignaling-independent manner, PD-1 suppressed PI3K activities triggered downstream of CXCR5. This inhibition was induced by PD-1 engagement by PD-L1 expressed on follicular bystander B cells. When unopposed by ICOS-mediated enhancement of PI3K activation, PD-1-mediated inhibition prevented follicular recruitment of activated T cells. Therefore, at the T-B border, PD-1 and PD-L1 favor follicular recruitment of those T cells expressing a high level of ICOS. By examining Tfh cells genetically impaired in PD-1 upregulation, we further found that PD-1 was also required for optimal GC localization of Tfh cells, for IL-21 production, and for setting a sufficiently stringent threshold for the GC B cell competition. Thus, by controlling both proper positioning and helper functions of Tfh cells, the inhibitory receptor PD-1 plays an essential role during the germinal center response.

RESULTS

PD-1 Suppresses PI3K Activation and Follicular T Cell Recruitment

It has previously been shown that ICOS promotes PI3K-dependent T cell motility and is required to drive follicular T cell recruitment in a costimulation-independent manner (Xu et al., 2013). Using the same quantitative *in vivo* homing assay as utilized in the previous study, we further verified that an intact p85-recruiting YxxM motif was required for ICOS-mediated promotion of follicular recruitment (Figure S1). Because lymphocyte motility

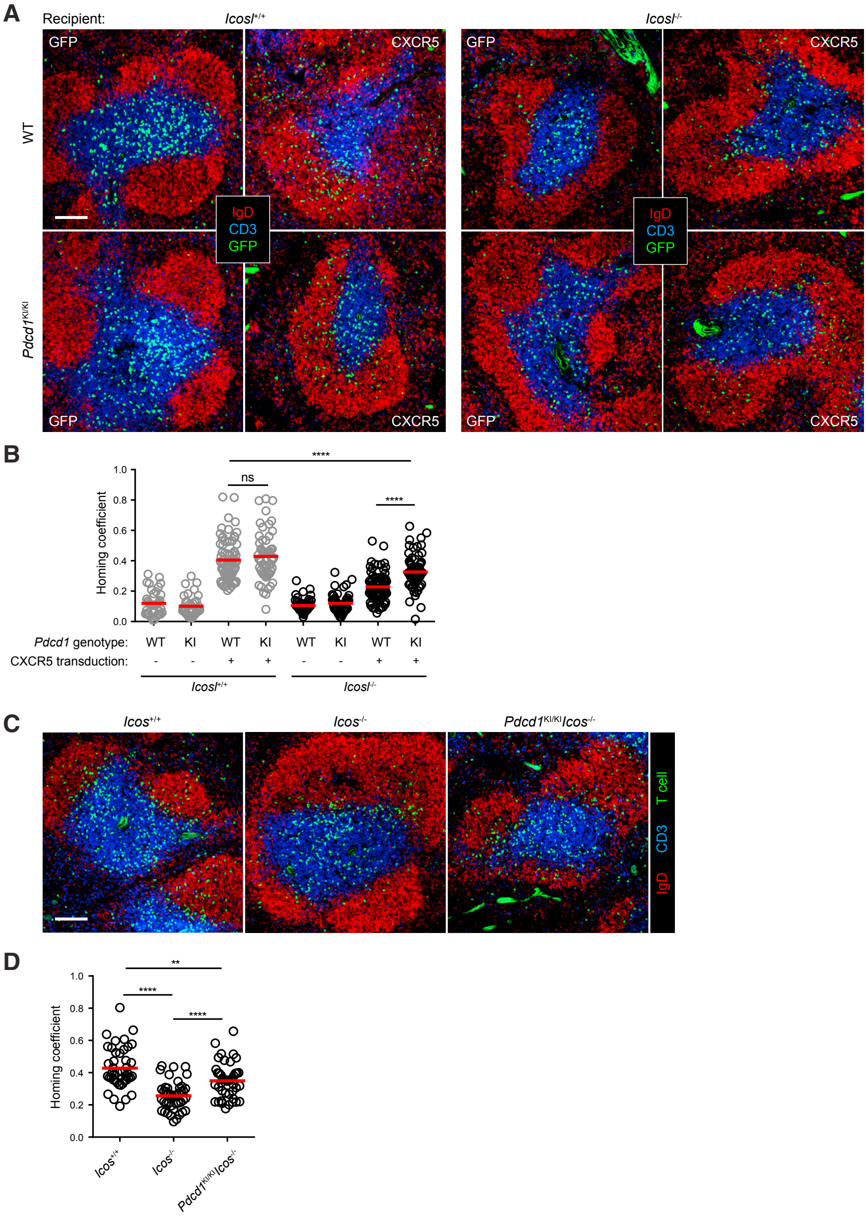

Figure 2. PD-1 Abrogation on Activated T Cells Rescues Follicular Homing Defect due to ICOSL-ICOS Deficiency

(A and B) CXCR5- or GFP-transduced wild-type (top) or *Pdcd1*$^{KI/KI}$ (bottom) CD4$^+$ T cells were transferred into *Icosl*$^{+/+}$ (left) or *Icosl*$^{-/-}$ (right) mice (2–3 × 10^6 cells per mouse). Representative splenic distribution patterns (A) and homing coefficients (B) of T cells 24 hr after adoptive transfer.

(C and D) Representative splenic distribution patterns (C) and homing coefficients (D) of CXCR5-transduced *Icos*$^{+/+}$, *Icos*$^{-/-}$, or *Pdcd1*$^{KI/KI}$*Icos*$^{-/-}$ CD4$^+$ T cells 24 hr after being adoptive transfer into B6 mice.

Each symbol denotes one section. Data are pooled from five (B) or three (D) independent experiments. Scale bar, 100 μm. **$p < 0.01$; ****$p < 0.0001$; ns, not significant.

in general requires PI3K activities and because signaling of chemokine receptors such as CXCR5 activates PI3K, it is puzzling why ICOS is additionally required for motility and recruitment of activated T cells into the follicle, whereas ICOS is not necessary for T cell migration in the T cell zone (Xu et al., 2013). Potentially, the follicular niche may contain factors that can inhibit T cell PI3K activities downstream of chemokine receptors such as CXCR5 and thereby impose a requirement for bystander ICOS signaling at the T-B border and in the follicle to boost and maintain PI3K activities and thereby promote T cell motility and recruitment. To test this hypothesis, we searched for surface-bound ligand-receptor pairs that meet the following three conditions. The receptor and ligand are expressed respectively by antigen-activated T cells and follicular parenchyma-constituting bystander B cells. Signaling through such receptors into T cells suppresses PI3K activities triggered by chemokine receptor CXCR5 and by ICOS. When this ligand or receptor is ablated, the requirement for ICOS to promote follicular migration may be relaxed.

Because PD-L1 is constitutively expressed by follicular B cells (Figure 1A), we first tested its effect on PI3K activation triggered by CXCR5 on T cells. To ensure a uniform response, T cells were retrovirally transduced with CXCR5 and PD-1 before being stimulated with CXCL13 in the presence of PD-L1-Fc fusion protein. As shown in Figure 1B, engagement of PD-1 by PD-L1-Fc protein significantly reduced CXCL13-triggered PI3K activities as measured by Akt phosphorylation. Consistent with this PI3K suppression, CXCL13-induced T cell polarization, which is a prerequisite for cell motility, was impaired when PD-1 was engaged by PD-L1-Fc (Figure S2). PD-L1-Fc treatment also inhibited ICOS-stimulated PI3K activities (Figure 1C). To test whether PD-1 can inhibit CXCR5-driven follicular migration, localization of CXCR5-, PD-1-transduced T cells (Figure 1D) were examined 24 hr after being transferred into naive, unimmunized mice. As shown in Figure 1E, significantly fewer PD-1-overexpressing T cells migrated into the follicle despite enforced CXCR5 expression, resulting in a reduced homing coefficient (Figure S3A).

Endogenous PD-1 Antagonizes ICOS and Limits Follicular Recruitment in the Bystander Mode

CD4$^+$ T cells upregulate PD-1 expression very soon after antigen stimulation (Chen et al., 2015; Keir et al., 2008). To test whether endogenously expressed PD-1 suppresses follicular T cell recruitment and, if so, whether such suppression underlies the requirement for bystander ICOS-ICOSL interactions for recruitment, we resorted to a PD-1 knock-in strain in which an

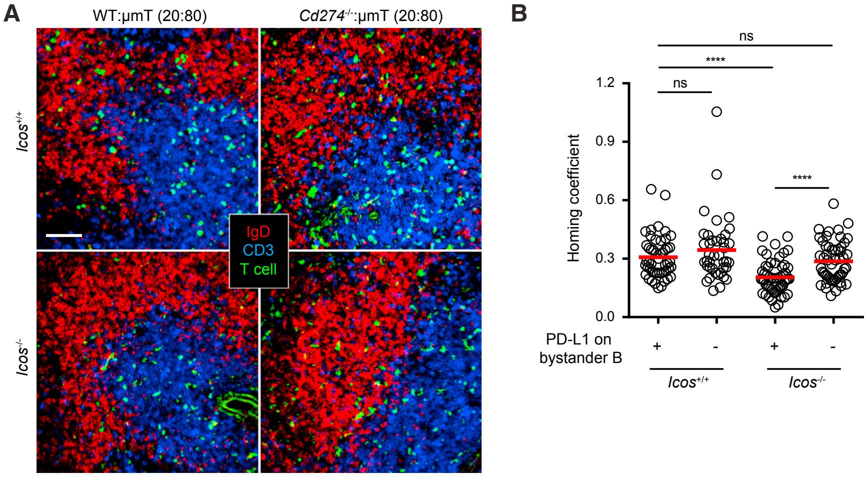

Figure 3. PD-L1 on Bystander B Cells Suppresses T Cell Recruitment

Splenic distribution patterns (A) and homing coefficients (B) of CXCR5-transduced *Icos*$^{+/+}$ (top) or *Icos*$^{-/-}$ (bottom) T cells 24 hr after adoptive transfer into mixed bone-marrow chimeric hosts of indicated types (2–3 × 10^6 cells per mouse). Each symbol denotes one tissue section. Data are pooled from three experiments. Scale bar, 50 μm. ***p < 0.001; ****p < 0.0001; ns, not significant.

AP-1-binding site in the *Pdcd1* promoter is disabled so that T cells homozygous for this mutation (*Pdcd1*$^{KI/KI}$) cannot upregulate PD-1 (Figure S4; Xiao et al., 2012). As exemplified in Figure 2A and quantitated in Figure 2B, whereas CXCR5-transduced *Pdcd1*$^{+/+}$ T cells efficiently migrated into follicles of normal B6 mice but failed to do so in *Icosl*$^{-/-}$ mice, as expected according to previous observations (Xu et al., 2013), *Pdcd1*$^{KI/KI}$ T cells were indeed able to migrate into follicles composed of not only wild-type but also *Icosl*$^{-/-}$ bystander B cells (Figure S3B). Furthermore, as compared to *Icos*$^{-/-}$ T cells, *Pdcd1*$^{KI/KI}$*Icos*$^{-/-}$ T cells exhibited a significantly improved ability to migrate into normal follicles (Figures 2C, 2D, and S3C). Importantly, because all the recipient mice used in these experiments were not immunized and transferred T cells are not stimulated by antigen *in vivo*, the inhibitory PD-1 effect on follicular T cell recruitment is likely based on triggering of PD-1 in a bystander mode by the ensemble of follicular B cells.

To validate the bystander mode of PD-1 activation in the follicle, by a CRISPR/Cas9 strategy we created a PD-L1 (encoded by *Cd274*)-deleted strain (Figure S5). We then made *Cd274*$^{-/-}$:μMT 20:80 bone-marrow (BM) chimera in which 80% BM cells are from the μMT background that cannot produce B cells and 20% BM cells are of the *Cd274*$^{-/-}$ genotype. Therefore, in such chimeras PD-L1 is absent from all follicular B cells while other hematopoietic cells are largely normal. When CXCR5-transduced *Icos*$^{-/-}$ T cells were transferred into control *Cd274*$^{+/+}$:μMT chimera, they failed to migrate into the follicle as efficiently as their wild-type counterpart T cells (Figure 3). However, this defect was significantly rescued in the *Cd274*$^{-/-}$:μMT chimera (Figures 3 and S3D). These data demonstrate that PD-1-mediated suppression of follicular recruitment is indeed mediated by PD-L1 expressed on bystander follicular B cells.

Bystander PD-1 Triggering Inhibits Follicular T Cell Recruitment via Its ITSM and Activates SHP-2

In the cytoplasmic domain of the PD-1 molecule, there are two inhibitory signaling motifs: Y225-based ITIM motif and Y248-based ITSM motif. When PD-1 functions as a co-inhibitory receptor to dampen TCR signaling, its ITSM plays the dominant role and mediates SHP-2 activation and recruitment (Chemnitz et al., 2004; Sheppard et al., 2004). To test whether bystander activation of PD-1 signals through ITIM or ITSM when inhibiting the follicular T cell recruitment, we compared Y225F mutant and Y248F mutant form of PD-1 molecules in the homing assay. As shown in Figure 4A and quantitated in Figure 4B, whereas the PD-1^{Y225F} mutant molecule was as inhibitory as the wild-type PD-1 molecule, PD-1^{Y248F} mutant lost the ability to suppress follicular T cell recruitment, indicating that ITSM also predominantly mediates PD-1 signaling activated in the bystander mode (Figure S3E). When activated T cells were stimulated *in vitro* by PD-L1-Fc, we also detected an increase in SHP2 phosphorylation, which was not affected by concomitant ICOS stimulation (Figure 4C). It is therefore likely that SHP2 plays a role in mediating bystander PD-1 signaling as well.

PD-1 Suppresses Overall Tfh Cell Development

Although the foregoing observations reveal a bystander mode of PD-1 functions and explains why bystander ICOS activation is required to promote follicular T cell recruitment, it remains paradoxical why Tfh cells must express a high level of PD-1 molecules. Consistent with previous findings of a negative role for the PD-1-PD-L1 molecular pair in humoral immunity (Hams et al., 2011), PD-1-overexpressing T cells exhibited a ~50% reduction in Tfh cell development, as measured by the frequency of CXCR5^{hi} or CXCR5^{hi}Bcl-6^{hi} cells (Figure S6A) or by the ratio between the follicular T cell density and their density in the T cell zone (Figure S6B). Combined with a ~70% reduction in T cell expansion (Figure S6A), the absolute number of Tfh cells would be reduced to 10%–15% of the normal level. Conversely, when *Pdcd1*$^{KI/KI}$ T cells that could not upregulate PD-1 were activated *in vivo* after antigen immunization, there was a 2- to 3-fold increase in Tfh cell development as measured by the frequency of CXCR5^{hi} or CXCR5^{hi}Bcl-6^{hi} cells (Figure S6C) or by the differential distribution between the follicle and the T cell zone (Figure S6D). Combined with a ~3-fold increase in T cell expansion (Figure S6C), there was typically a 6- to 10-fold increase in the absolute number of Tfh cells. Therefore, by these criteria widely used in the literature to evaluate Tfh cell development, PD-1 is arguably one of the most potent suppressors of Tfh cell development.

Bystander Engagement of PD-1 Helps Tfh Cell Concentration in GCs

To resolve this conceptual paradox, we tested the possibility that heightened PD-1 expression actually serves positive functions for Tfh cells. First, we confirmed previous findings that PD-1 was required for optimal IL-21 production by Tfh cells

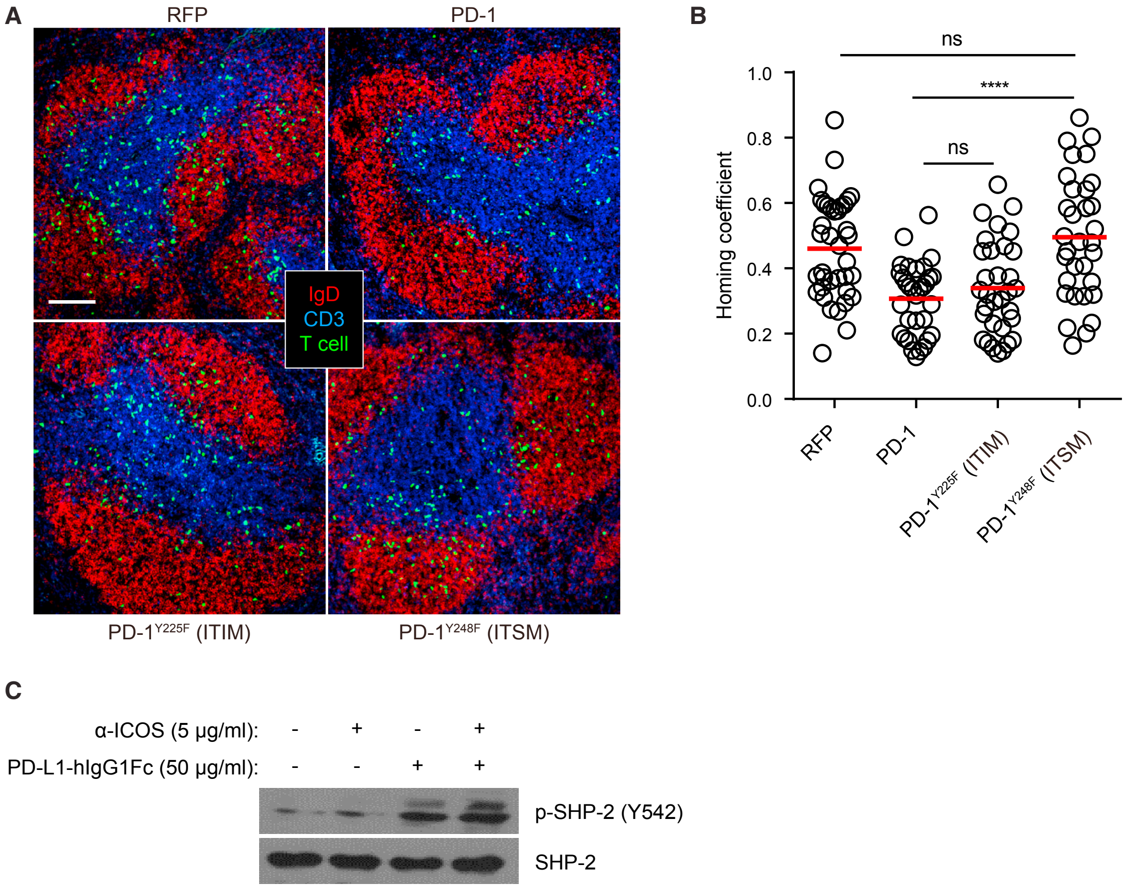

Figure 4. PD-1-Mediated Suppression of Follicular T Cell Recruitment Implicates ITSM and SHP-2

(A) Splenic distribution patterns of $CD4^+$ T cells that were co-transduced with a vector expressing CXCR5 and another vector expressing control RFP (top left) or wild-type PD-1 (top right) or ITIM-mutated PD-1^{Y225F} (bottom left) or ITSM-mutated PD-$1Y^{248F}$ (bottom right) 24 hr after being transferred into B6 recipients (2–3 × 10^6 per mouse).

(B) Scatterplots of homing coefficients of the four groups in (A). Each symbol denotes one tissue section. Data are pooled from three experiments. Scale bar, 100 μm. ****$p < 0.0001$; ns, not significant.

(C) SHP-2 phosphorylation in $CD4^+$ T cells after 30 min anti-ICOS stimulation in the presence or absence of PD-L1-Fc. Cells were pre-starved for 3 hr. Data represent two independent experiments.

(Figures S7A–S7C; Good-Jacobson et al., 2010; Kawamoto et al., 2012). Next, given that Tfh cells must be concentrated in GCs in order to properly function as B cell helpers and that PD-1 is mainly a marker for GC-resident Tfh cells (Haynes et al., 2007), we considered the possibility that heightened PD-1 expression could help concentrate T cells into GCs. This is because when the overall chemo-sensing program of $CXCR5^+CCR7^{lo}S1PR2^+$ Tfh cells favors localization to the follicular center, PD-1-mediated motility inhibition due to bystander PD-L1 engagement in the follicular mantle could push these cells into the GC. To test this scenario, we transferred wild-type MD4 B cells that recognize hen egg lysozyme (HEL) and *Pdcd1*$^{KI/KI}$ or wild-type OT-II T cells that recognize ovalbumin$(OVA)_{323-339}$ presented on the I-A^b molecule into B6 mice. Following HEL-OVA immunization, we examined the distribution of OT-II Tfh cells in GCs and the follicular area. Consistent with a PD-1-mediated concentrating effect, we indeed observed a decrease in the density ratio between the GC and the follicle for *Pdcd1*$^{KI/KI}$ Tfh cells as compared to that for their wild-type counterparts (Figures 5A and 5B). We also examined the distribution of wild-type and *Pdcd1*$^{KI/KI}$ Tfh cells in wild-type MD4 GCs developed in PD-L1-sufficient or -deficient mice. As exemplified in Figure 5C and quantitated in Figure 5D, loss of either PD-1 on Tfh cells or PD-L1 on follicular bystander B cells significantly reduced the Tfh cell density in GCs in comparison to that in the follicle. These data suggest that PD-1 and PD-L1 serve to concentrate Tfh cells into the GC from the follicle.

PD-1 Concentrates T Cells to GCs by Limiting CXCR3 Distraction

While PD-1 can optimize follicle-to-GC partitioning of Tfh cells by directly suppressing follicular migration in a bystander mode, its signals may also change intrinsic properties of Tfh cells to optimize their GC positioning indirectly. To probe this issue, we co-transferred MD4 B cells and wild-type or *Pdcd1*$^{KI/KI}$ OT-II cells into separate B6 mice, immunized these mice with HEL-OVA, and isolated two types of Tfh cells after MD4 GCs developed for transcriptome analysis by RNA-seq. Using $p_{adj} < 0.01$ as the cutoff, we observed only a few genes differentially

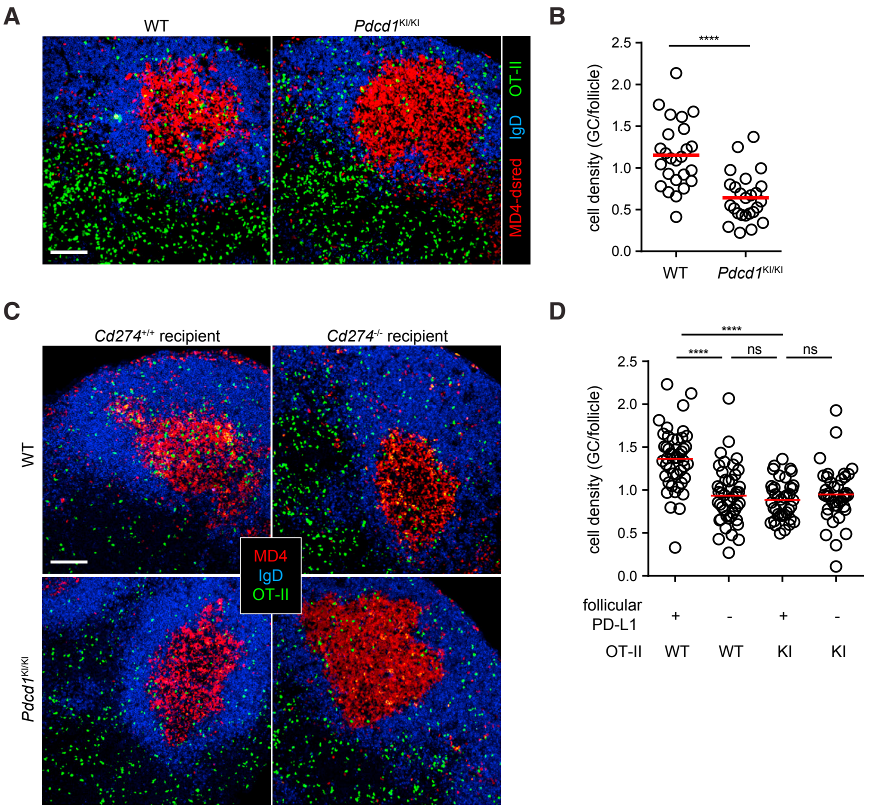

Figure 5. PD-1 and PD-L1 Promote Tfh Cell Concentration toward the GC Area

(A and B) GFP-transduced wild-type or *Pdcd1*$^{KI/KI}$ OT-II T cells were transferred into B6 mice (5 × 10^5 sorted GFP$^+$ cells per mouse) together with 5 × 10^5 dsRed-expressing MD4 B cells. OT-II distribution patterns (A) and density ratios between the GCs and the follicle (B) in draining lymph nodes 5 days after subcutaneous HEL-OVA immunization.

(C and D) Wild-type or *Cd274*$^{-/-}$ mice received 5 × 10^5 GFP-transduced wild-type or *Pdcd1*$^{KI/KI}$ OT-II T cells together with 5 × 10^5 dsRed-expressing MD4 B cells. OT-II distribution patterns (C) and density ratios between the GCs and the follicle (D) in draining lymph nodes 5 days after subcutaneous HEL-OVA immunization.

Scale bar, 100 μm. All data are pooled from three independent experiments involving 3–4 mice per condition per experiment. ****$p < 0.0001$; ns, not significant.

expressed by CXCR5^{hi} Tfh cells of the two genotypes (Figure 6A; see Table S1 for a full list), including *Pdcd1* and *Cxcr3*. CXCR3, expressed by T cells after TCR stimulation (Nakajima et al., 2002), is the receptor for chemokine CXCL9 and CXCL10 that are highly expressed in interfollicular regions immediately outside of follicles (Groom et al., 2012). Responsiveness to CXCL9 or CXCL10 is not conducive to GC localization and incompatible with a Tfh cell phenotype (Campbell et al., 2001). Consistent with a requirement for PD-1 to dampen CXCR3 expression to limit potential distraction of Tfh cells by CXCL9 and/or CXCL10, we found heightened CXCR3 expression by *Pdcd1*$^{KI/KI}$ Tfh cells as compared to wild-type Tfh cells (Figure 6B). To test whether CXCR3 can indeed distract Tfh cells from GC localization, we targeted CXCR3 by two separate shRNAs (Figure 6C). As exemplified in Figure 6D and quantitated in Figure 6E, these two independent CXCR3-targeting shRNAs were both able to significantly improve the follicle-to-GC partitioning for both the wild-type and *Pdcd1*$^{KI/KI}$ Tfh cells. Together, these data indicate that a positive function of PD-1 expression is to restrict CXCR3-mediated distraction and promote Tfh cell concentration in the GC region.

PD-1 Maintains the Stringency of GC Selection

Another aspect of GC biology that PD-1 on Tfh cells may regulate in a positive manner is affinity selection. When functioning as a co-inhibitory receptor engaged by its ligands on antigen-presenting cells, PD-1 can dampen TCR signaling (Yokosuka et al., 2012; Zinselmeyer et al., 2013) and thereby reduce the ligand sensitivity of Tfh cells, a condition that should increase the overall stringency of selection in GCs (Wang et al., 2016). To test this hypothesis, we first validated that, as would be predicted by the overall increase in *Pdcd1*$^{KI/KI}$ Tfh cell development (Figure S6), selective removal of PD-1 from adoptively transferred OT-II T cells would exaggerate the GC response following NP-OVA immunization in *Sap*$^{-/-}$ mice (Figures S7D and S7E), which cannot endogenously generate competent Tfh cells (Qi et al., 2008; Yusuf et al., 2010). Of note, total plasma cells were also increased at the peak of the immune response. More importantly, the NP-binding fraction in GCs was significantly reduced in the *Pdcd1*$^{KI/KI}$ OT-II group following NP-OVA immunization (Figures S7D and S7E), implying a reduced stringency of GC selection and outgrowth of low-affinity or irrelevant specificities (Linterman et al., 2011).

Next, we constructed 1:1 BM chimera using CD45.1 mixed with *Cd274*$^{-/-}$ CD45.2 BM cells or CD45.1 mixed with *Cd274*$^{+/+}$ CD45.2 BM cells. As shown in Figures 7A and 7B, at day 8, 13, and 21 after NP-KLH immunization, *Cd274*$^{-/-}$ B cells dominated the GC, the memory (except for day 21), and the splenic plasma cell compartments. PD-L1-deficient B cells also tended to dominate the BM plasma cell compartment as measured on day 21. These data suggest that the absence of PD-L1 allowed individual B cells to acquire more T cell help. On the other hand, PD-L1-deficient B cells were outcompeted by wild-type CD45.1 cells within NP-binding GC B cells (Figures 7A and 7B), suggestive of lower NP-binding affinities of PD-L1-deficient B cells and/or outgrowth of irrelevant antigen specificities. To directly examine the frequency of high-affinity cells within the mixed GCs, we sorted NP-binding GC B cells and sequenced the canonical V_H186.2 gene segment. As shown in Figure 7C, PD-L1-deficient B cells harbored significantly fewer affinity-improving W33L mutation, despite a similar overall frequency of mutation. Together, these data indicate that PD-1 and PD-L1 interactions between Tfh and GC B cells help to maintain the stringency of affinity-based selection.

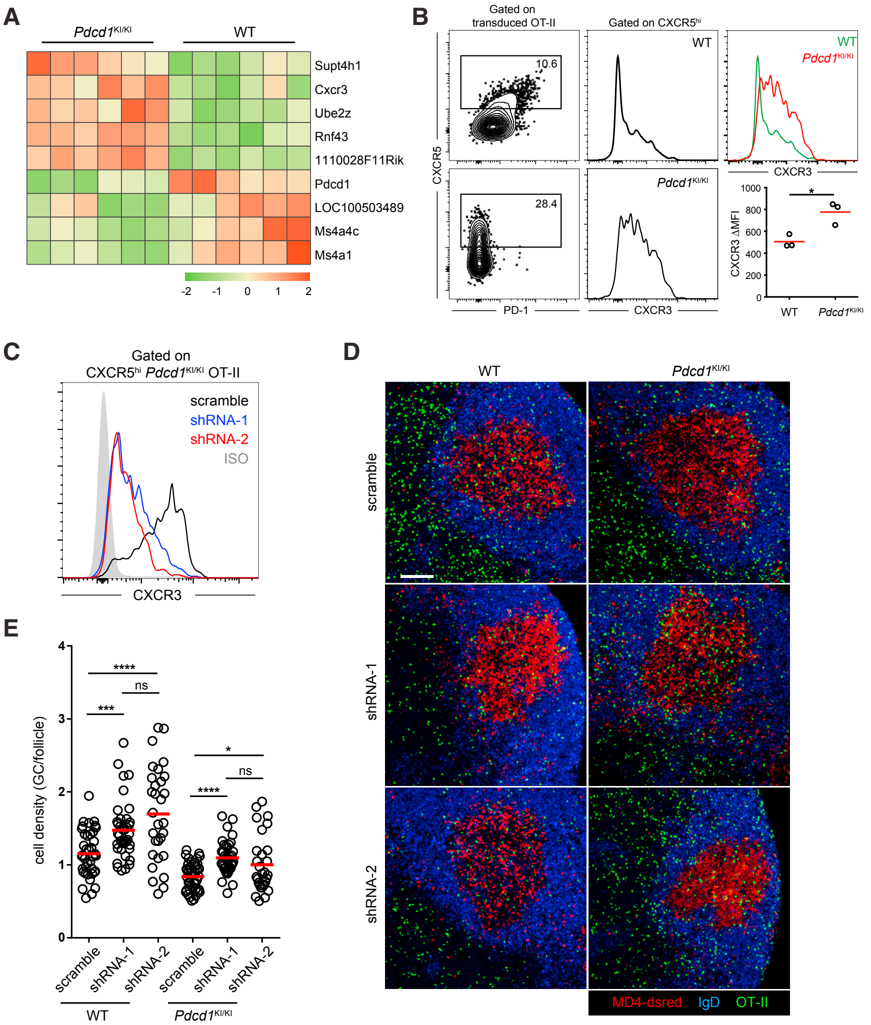

Figure 6. PD-1 Restricts CXCR3 Expression and Promotes Confinement of Tfh Cells in GCs

(A and B) GFP-transduced wild-type or *Pdcd1*$^{KI/KI}$ OT-II T cells were transferred into B6 mice (5 × 10^{5} sorted GFP^{+} cells per mouse) together with 5 × 10^{5} dsRed-expressing MD4 B cells, and the recipients were subcutaneously immunized with HEL-OVA.

(A) CXCR5^{hi} OT-II T cells were sorted from the two groups on day 5 and subjected to transcriptomic analysis by RNA-seq. Shown is unsupervised clustering of genes differentially expressed as defined by an adjusted p value < 0.01.

(B) CXCR3 expression on CXCR5^{hi} wild-type and *Pdcd1*$^{KI/KI}$ OT-II T cells. Left, CXCR5^{hi} gate; middle, histograms; right, representative CXCR3 histogram overlay and MFI values of CXCR5^{hi} OT-II cells in three mice; data representative of three independent experiments; *p < 0.05.

(C) CXCR3 expression on CXCR5^{hi} *Pdcd1*$^{KI/KI}$ OT-II T cells that were transduced with scramble control shRNA or two separate CXCR3-targeting shRNAs. Grey histogram, isotype control.

(D and E) Wild-type (left) or *Pdcd1*$^{KI/KI}$ (right) OT-II T cells were transduced with scramble control shRNA or CXCR3-targeting shRNA and then were transferred into B6 mice (5 × 10^{5} Ametrine^{+} transduced cells per mouse) together with 5 × 10^{5} dsRed-expressing MD4 B cells. OT-II distribution patterns (D) and density ratios between the GCs and the follicle (E) in draining lymph nodes 5 days after subcutaneous HEL-OVA immunization.

Scale bar, 100 μm. Each symbol denotes one tissue section. Data are pooled from three experiments involving three and four mice per condition per experiment. ns, not significant; *p < 0.05; ****p < 0.0001.

DISCUSSION

This study uncovers a bystander mode of PD-1 function in suppressing the follicular recruitment of activated helper T cells. It is based on PD-1 engagement by PD-L1 constitutively expressed on follicular naive B cells. This is reminiscent of previous findings that ICOS functions in a bystander mode to optimize T cell motility and to promote follicular recruitment of activated helper T cells (Xu et al., 2013). Given heightened CXCR5 expression on Tfh cells, CXCL13 in the follicle could be sufficient in driving their PI3K activation and motility, if not because of the inhibitory PD-L1 field. Indeed, the PD-1-mediated inhibition is likely the main reason why bystander ICOS activation is required for optimal T cell recruitment. Noticeably, the follicular homing defect of ICOS-deficient T cells is only partially rescued by abrogation of bystander PD-1-PD-L1 interactions, suggesting that additional inhibitory pathways may be in play to suppress follicular recruitment.

It is striking that both ICOS and PD-1 can function independently of TCR stimulation, that both regulate PI3K activities, and that ligands of both receptors are constitutively expressed by follicular bystander B cells. There is also evidence that CTLA-4, another B7-family molecule, may directly influence T cell adhesion and migration (Schneider et al., 2005). Possibly, in addition to their classical functions of co-signaling with the TCR, all B7-family molecules may possess an ability to directly regulate T cell motility and positioning in different settings *in vivo*.

A benefit of PD-1-mediated inhibition of follicular recruitment would be to ensure cells that actually move and dwell in the follicle are those that highly express ICOS. At the same time, because ICOS costimulation also promotes PD-1 upregulation (Keir et al., 2008), TCR triggering and ICOS costimulation by cognate B cells in the follicle would promote and maintain PD-1 expression on Tfh cells. As a result, follicle-resident T cells could be locked in an ICOS^{hi}PD-1^{hi} state. An ICOS^{hi}PD-1^{hi} state is important for specific helper functions of Tfh cells. First, both ICOS and PD-1 promote IL-21 production from T cells. ICOS has been shown to induce c-Maf and IL-21 expression in both Tfh and Th17 cells (Bauquet et al., 2009). With this study included, PD-1 has been shown in three independent systems as required for optimal IL-21 production from Tfh cells, providing one probable reason for why Tfh cells need to express high levels

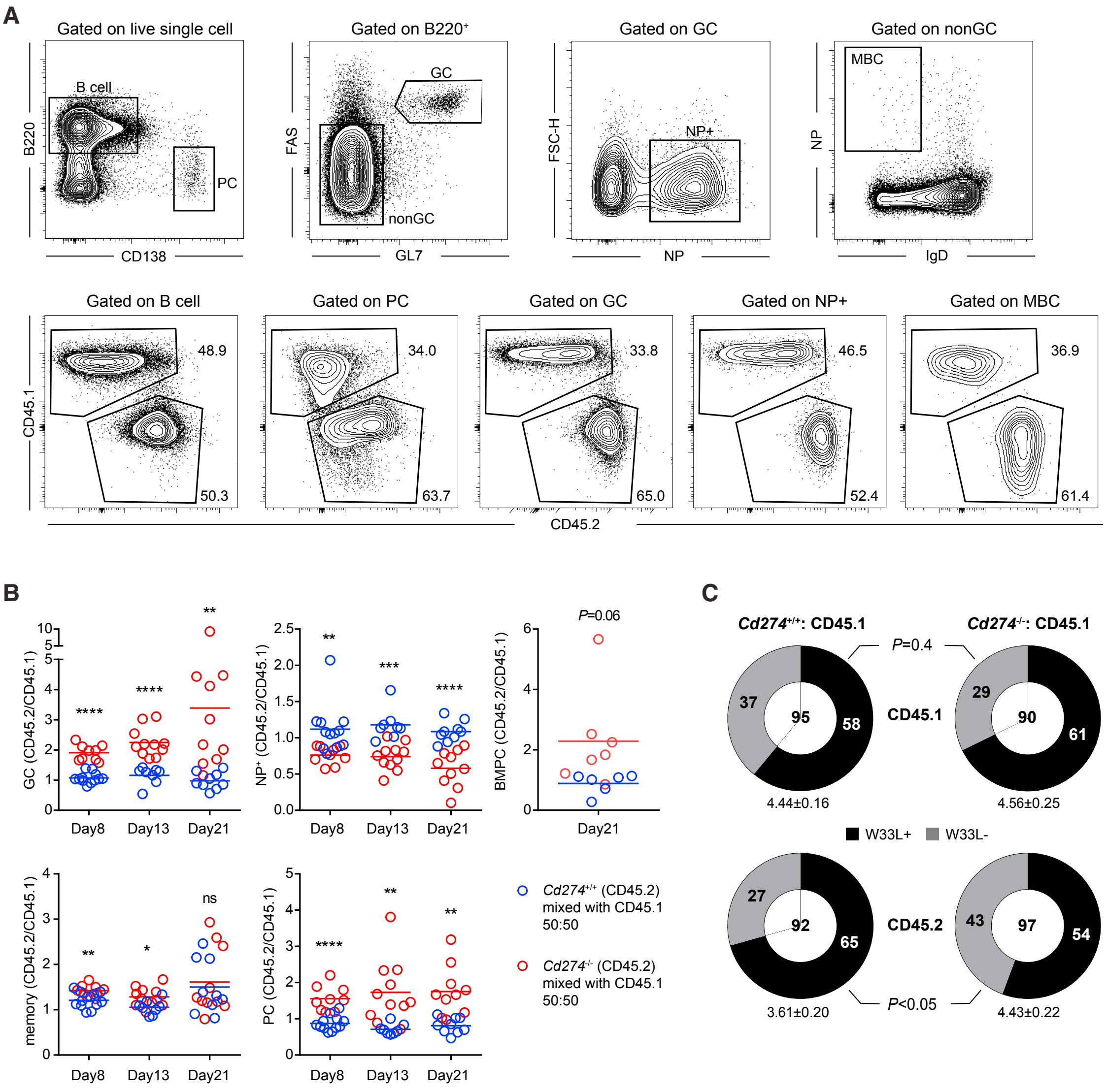

Figure 7. Compromised Affinity Maturation in the Absence of PD-1-PD-L1 Interactions between GC Tfh and B Cells

Mixed BM chimeras were constructed using 50% CD45.2 *Cd274*$^{+/+}$ or *Cd274*$^{-/-}$ BM cells and 50% CD45.1 BM cells. Chimeric mice were immunized with NP-KLH.

(A) Gating strategies for total B cells, plasma cells, GC B cells, NP-specific GC B cells, and memory B cells (top row) and for CD45.1 and CD45.2 cells within each indicated population (bottom row). The example is from *Cd274*$^{-/-}$:CD45.1 chimera.

(B) Scatterplots of competitive competencies of CD45.2 cells in the two sets of mixed chimeras contributing to GC B cells, NP-specific GC B cells, NP-specific memory B cells and plasma cells, and BM plasma cells at day 8, day 13, or day 21 after immunization. The competitive competency is defined as the CD45.2/CD45.1 ratio in the indicated compartment normalized against the same ratio in the total B cell compartment or the GC compartment in the case of NP-specific GC cells. Data are pooled from three independent experiments involving 3–4 mice per group per experiment. Each symbol denotes one mouse. *$p < 0.05$; **$p < 0.01$; ***$p < 0.001$; ****$p < 0.0001$; ns, not significant.

(C) Total mutation rates and frequencies of affinity-enhancing W33L mutation in NP-specific, V_H186.2-carrying GC B cells isolated on day 13 after NP-KLH immunization. Numbers in the center of the pie chart indicate total numbers of clones analyzed, and numbers of W33L$^+$ or W33L$^-$ cells are indicated. Numbers of total mutation are presented as mean ± SEM. Frequencies of W33L mutations were compared by the Fisher's exact test. Data are pooled from three independent experiments, each involving 3–4 mice for each group.

of PD-1 and be in the ICOS^{hi}PD-1^{hi} state. However, given the general antagonistic relationship between ICOS and PD-1 signaling, future studies will have to delineate how exactly PD-1 promotes IL-21 production. Second, by restricting CXCR3 expression and suppressing potential distraction from CXCL9 and/or CXCL10, PD-1 helps to concentrate Tfh cells in GCs. The fact that some Tfh cells still express CXCR3 is consistent with previous findings that these cells are in a dynamic flux between GCs and follicles with a potential to escape the follicular area entirely (Lu et al., 2017; Qi et al., 2008; Shulman et al., 2013; Suan et al., 2015). Finally, in the dynamic GC environment, the ICOS-ICOSL pair promote affinity maturation by establishing an intercellular positive-feedback loop between Tfh and GC B cells, ensuring that high-affinity mutants that stochastically

arise can be rapidly selected by T cells (Liu et al., 2015). PD-1 can reduce the TCR ligand sensitivity (Yokosuka et al., 2012) and thereby enforce a more stringent selection threshold for competing B cells to also promote affinity maturation. On the other hand, PD-1 and PD-L1 have been reported to stabilize the immunological synapse between CD8 T cells and viral antigen-presenting cells during chronic viral infection (Zinselmeyer et al., 2013). This cell type-specific complexity of PD-1 functions requires further mechanistic clarification. When PD-1, PD-L2, or PD-L1 together with PD-L2 were ablated from the germline, these knockout animals exhibited reduction in GC and NP-specific plasma cells and increase in serum antibody affinities after NP-CGG immunization (Good-Jacobson et al., 2010). Given that non-Tfh cells including follicular T regulatory cells and B cells can express PD-1 and that PD-1 has a more general role in maintaining peripheral tolerance (Keir et al., 2008; Sage et al., 2013), more complex physiology beyond Tfh-GC B cell interactions is probably also involved in producing those phenotypes.

Our study also highlights the importance of follicular bystander B cells, which have not been routinely considered as a relevant player in studies of cellular and molecular mechanisms regulating the Tfh cell development and germinal center reaction. Because bystander B cells that do not present antigen to Tfh cells are numerically dominant in the follicle even during active GC responses, their ensemble constitutes a niche that Tfh cells by definition cannot escape, and these T cells are constantly subjected to bystander triggering of ICOS, PD-1, and possibly additional receptors that would otherwise modulate TCR signaling in the context of cognate interactions between Tfh cells and antigen-specific B cells. From the perspective of niche regulation of motility and T cell recruitment, it is notable that many non-hematopoietic tissue cells can express PD-L1 under the influence of inflammatory cytokines such as interferons (Eppihimer et al., 2002; Schreiner et al., 2004) and that many types of tumors and tumor-associated macrophages can densely express PD-L1 (Gibbons Johnson and Dong, 2017; Kythreotou et al., 2017). We speculate that inhibitory effects of PD-1 on T cell migration may also be involved in regulating T cell infiltration into those inflammatory tissues and tumor niches.

STAR★METHODS

Detailed methods are provided in the online version of this paper and include the following:

- KEY RESOURCES TABLE
- CONTACT FOR REAGENT AND RESOURCE SHARING
- EXPERIMENT MODEL AND SUBJECT DETAILS
- METHOD DETAILS
  - Animals and immunizations
  - Construction of bone marrow chimeras
  - Construction of *Cd274*$^{-/-}$ mice
  - Retrovirus and *in vitro* transduction
  - Immunoblotting
  - Chemokine-stimulated T cell polarization assay
  - Follicular homing assay and immunohistochemistry
  - Flow cytometry
  - V_H186.2 sequence analysis of NP-specific GC B cells
  - CXCR3 knockdown
  - RNA-seq
- QUANTIFICATION AND STATISTICAL ANAYSIS
- DATA AND SOFTWARE AVAILABILITY

SUPPLEMENTAL INFORMATION

Supplemental Information includes seven figures and one table and can be found with this article online at https://doi.org/10.1016/j.immuni.2018.06.012.

ACKNOWLEDGMENTS

The authors thank Dr. P. Schwartzberg for providing the *Sap*$^{-/-}$ mice and Dr. Y.-C. Liu for the MSCV-LMP vector. This work was funded in part by the Ministry of Science and Technology "973" program (grant no. 2014CB542501), National Natural Science Foundation of China (grant no. 81330070, 81425011, 81621002), and the Tsinghua-Peking Center for Life Sciences. This work was also funded in part by the Bill & Melinda Gates Foundation and the Howard Hughes Medical Institute. The findings and conclusions within are those of the authors and do not necessarily reflect positions or policies of the Bill & Melinda Gates Foundation or the Howard Hughes Medical Institute.

AUTHOR CONTRIBUTIONS

J.S. conducted a majority of the experiments, and all authors contributed to data interpretation and experimental design. H.Q. supervised the work and wrote the paper with J.S.

DECLARATION OF INTEREST

The authors declare no conflicts of interest.

Received: December 19, 2017
Revised: May 7, 2018
Accepted: June 22, 2018
Published: July 31, 2018

REFERENCES

Ballesteros-Tato, A., León, B., Graf, B.A., Moquin, A., Adams, P.S., Lund, F.E., and Randall, T.D. (2012). Interleukin-2 inhibits germinal center formation by limiting T follicular helper cell differentiation. Immunity *36*, 847–856.

Barber, D.L., Wherry, E.J., Masopust, D., Zhu, B., Allison, J.P., Sharpe, A.H., Freeman, G.J., and Ahmed, R. (2006). Restoring function in exhausted CD8 T cells during chronic viral infection. Nature *439*, 682–687.

Bauquet, A.T., Jin, H., Paterson, A.M., Mitsdoerffer, M., Ho, I.C., Sharpe, A.H., and Kuchroo, V.K. (2009). The costimulatory molecule ICOS regulates the expression of c-Maf and IL-21 in the development of follicular T helper cells and TH-17 cells. Nat. Immunol. *10*, 167–175.

Campbell, D.J., Kim, C.H., and Butcher, E.C. (2001). Separable effector T cell populations specialized for B cell help or tissue inflammation. Nat. Immunol. *2*, 876–881.

Chemnitz, J.M., Parry, R.V., Nichols, K.E., June, C.H., and Riley, J.L. (2004). SHP-1 and SHP-2 associate with immunoreceptor tyrosine-based switch motif of programmed death 1 upon primary human T cell stimulation, but only receptor ligation prevents T cell activation. J. Immunol. *173*, 945–954.

Chen, X., Ma, W., Zhang, T., Wu, L., and Qi, H. (2015). Phenotypic Tfh development promoted by CXCR5-controlled re-localization and IL-6 from radiation-resistant cells. Protein Cell *6*, 825–832.

Choi, Y.S., Kageyama, R., Eto, D., Escobar, T.C., Johnston, R.J., Monticelli, L., Lao, C., and Crotty, S. (2011). ICOS receptor instructs T follicular helper cell versus effector cell differentiation via induction of the transcriptional repressor Bcl6. Immunity *34*, 932–946.

Crotty, S. (2011). Follicular helper CD4 T cells (T_{FH}). Annu. Rev. Immunol. *29*, 621–663.

Czar, M.J., Kersh, E.N., Mijares, L.A., Lanier, G., Lewis, J., Yap, G., Chen, A., Sher, A., Duckett, C.S., Ahmed, R., and Schwartzberg, P.L. (2001). Altered lymphocyte responses and cytokine production in mice deficient in the X-linked lymphoproliferative disease gene SH2D1A/DSHP/SAP. Proc. Natl. Acad. Sci. USA *98*, 7449–7454.

Deenick, E.K., Chan, A., Ma, C.S., Gatto, D., Schwartzberg, P.L., Brink, R., and Tangye, S.G. (2010). Follicular helper T cell differentiation requires continuous antigen presentation that is independent of unique B cell signaling. Immunity *33*, 241–253.

Eppihimer, M.J., Gunn, J., Freeman, G.J., Greenfield, E.A., Chernova, T., Erickson, J., and Leonard, J.P. (2002). Expression and regulation of the PD-L1 immunoinhibitory molecule on microvascular endothelial cells. Microcirculation *9*, 133–145.

Gibbons Johnson, R.M., and Dong, H. (2017). Functional expression of programmed death-ligand 1 (B7-H1) by immune cells and tumor cells. Front. Immunol. *8*, 961.

Goenka, R., Barnett, L.G., Silver, J.S., O'Neill, P.J., Hunter, C.A., Cancro, M.P., and Laufer, T.M. (2011). Cutting edge: dendritic cell-restricted antigen presentation initiates the follicular helper T cell program but cannot complete ultimate effector differentiation. J. Immunol. *187*, 1091–1095.

Good-Jacobson, K.L., Szumilas, C.G., Chen, L., Sharpe, A.H., Tomayko, M.M., and Shlomchik, M.J. (2010). PD-1 regulates germinal center B cell survival and the formation and affinity of long-lived plasma cells. Nat. Immunol. *11*, 535–542.

Groom, J.R., Richmond, J., Murooka, T.T., Sorensen, E.W., Sung, J.H., Bankert, K., von Andrian, U.H., Moon, J.J., Mempel, T.R., and Luster, A.D. (2012). CXCR3 chemokine receptor-ligand interactions in the lymph node optimize CD4+ T helper 1 cell differentiation. Immunity *37*, 1091–1103.

Hams, E., McCarron, M.J., Amu, S., Yagita, H., Azuma, M., Chen, L., and Fallon, P.G. (2011). Blockade of B7-H1 (programmed death ligand 1) enhances humoral immunity by positively regulating the generation of T follicular helper cells. J. Immunol. *186*, 5648–5655.

Harker, J.A., Lewis, G.M., Mack, L., and Zuniga, E.I. (2011). Late interleukin-6 escalates T follicular helper cell responses and controls a chronic viral infection. Science *334*, 825–829.

Haynes, N.M., Allen, C.D., Lesley, R., Ansel, K.M., Killeen, N., and Cyster, J.G. (2007). Role of CXCR5 and CCR7 in follicular Th cell positioning and appearance of a programmed cell death gene-1^{high} germinal center-associated subpopulation. J. Immunol. *179*, 5099–5108.

Hui, E., Cheung, J., Zhu, J., Su, X., Taylor, M.J., Wallweber, H.A., Sasmal, D.K., Huang, J., Kim, J.M., Mellman, I., and Vale, R.D. (2017). T cell costimulatory receptor CD28 is a primary target for PD-1-mediated inhibition. Science *355*, 1428–1433.

Johnston, R.J., Poholek, A.C., DiToro, D., Yusuf, I., Eto, D., Barnett, B., Dent, A.L., Craft, J., and Crotty, S. (2009). Bcl6 and Blimp-1 are reciprocal and antagonistic regulators of T follicular helper cell differentiation. Science *325*, 1006–1010.

Johnston, R.J., Choi, Y.S., Diamond, J.A., Yang, J.A., and Crotty, S. (2012). STAT5 is a potent negative regulator of T_{FH} cell differentiation. J. Exp. Med. *209*, 243–250.

Kawamoto, S., Tran, T.H., Maruya, M., Suzuki, K., Doi, Y., Tsutsui, Y., Kato, L.M., and Fagarasan, S. (2012). The inhibitory receptor PD-1 regulates IgA selection and bacterial composition in the gut. Science *336*, 485–489.

Keir, M.E., Butte, M.J., Freeman, G.J., and Sharpe, A.H. (2008). PD-1 and its ligands in tolerance and immunity. Annu. Rev. Immunol. *26*, 677–704.

Kim, D., Pertea, G., Trapnell, C., Pimentel, H., Kelley, R., and Salzberg, S.L. (2013). TopHat2: accurate alignment of transcriptomes in the presence of insertions, deletions and gene fusions. Genome Biol. *14*, R36.

Kythreotou, A., Siddique, A., Mauri, F.A., Bower, M., and Pinato, D.J. (2017). Pd-L1. J. Clin. Pathol. *71*, 189–194.

Linterman, M.A., Pierson, W., Lee, S.K., Kallies, A., Kawamoto, S., Rayner, T.F., Srivastava, M., Divekar, D.P., Beaton, L., Hogan, J.J., et al. (2011). Foxp3^{+} follicular regulatory T cells control the germinal center response. Nat. Med. *17*, 975–982.

Liu, D., Xu, H., Shih, C., Wan, Z., Ma, X., Ma, W., Luo, D., and Qi, H. (2015). T-B-cell entanglement and ICOSL-driven feed-forward regulation of germinal centre reaction. Nature *517*, 214–218.

Love, M.I., Huber, W., and Anders, S. (2014). Moderated estimation of fold change and dispersion for RNA-seq data with DESeq2. Genome Biol. *15*, 550.

Lu, P., Shih, C., and Qi, H. (2017). Ephrin B1-mediated repulsion and signaling control germinal center T cell territoriality and function. Science *356*, 356.

McDonald, P.W., Read, K.A., Baker, C.E., Anderson, A.E., Powell, M.D., Ballesteros-Tato, A., and Oestreich, K.J. (2016). IL-7 signalling represses Bcl-6 and the TFH gene program. Nat. Commun. *7*, 10285.

Morita, S., Kojima, T., and Kitamura, T. (2000). Plat-E: an efficient and stable system for transient packaging of retroviruses. Gene Ther. *7*, 1063–1066.

Nakajima, C., Mukai, T., Yamaguchi, N., Morimoto, Y., Park, W.R., Iwasaki, M., Gao, P., Ono, S., Fujiwara, H., and Hamaoka, T. (2002). Induction of the chemokine receptor CXCR3 on TCR-stimulated T cells: dependence on the release from persistent TCR-triggering and requirement for IFN-gamma stimulation. Eur. J. Immunol. *32*, 1792–1801.

Nurieva, R.I., Chung, Y., Hwang, D., Yang, X.O., Kang, H.S., Ma, L., Wang, Y.H., Watowich, S.S., Jetten, A.M., Tian, Q., and Dong, C. (2008). Generation of T follicular helper cells is mediated by interleukin-21 but independent of T helper 1, 2, or 17 cell lineages. Immunity *29*, 138–149.

Nurieva, R.I., Chung, Y., Martinez, G.J., Yang, X.O., Tanaka, S., Matskevitch, T.D., Wang, Y.H., and Dong, C. (2009). Bcl6 mediates the development of T follicular helper cells. Science *325*, 1001–1005.

Nurieva, R.I., Podd, A., Chen, Y., Alekseev, A.M., Yu, M., Qi, X., Huang, H., Wen, R., Wang, J., Li, H.S., et al. (2012). STAT5 protein negatively regulates T follicular helper (Tfh) cell generation and function. J. Biol. Chem. *287*, 11234–11239.

Parry, R.V., Chemnitz, J.M., Frauwirth, K.A., Lanfranco, A.R., Braunstein, I., Kobayashi, S.V., Linsley, P.S., Thompson, C.B., and Riley, J.L. (2005). CTLA-4 and PD-1 receptors inhibit T-cell activation by distinct mechanisms. Mol. Cell. Biol. *25*, 9543–9553.

Qi, H. (2016). T follicular helper cells in space-time. Nat. Rev. Immunol. *16*, 612–625.

Qi, H., Cannons, J.L., Klauschen, F., Schwartzberg, P.L., and Germain, R.N. (2008). SAP-controlled T-B cell interactions underlie germinal centre formation. Nature *455*, 764–769.

Sage, P.T., Francisco, L.M., Carman, C.V., and Sharpe, A.H. (2013). The receptor PD-1 controls follicular regulatory T cells in the lymph nodes and blood. Nat. Immunol. *14*, 152–161.

Schneider, H., Valk, E., da Rocha Dias, S., Wei, B., and Rudd, C.E. (2005). CTLA-4 up-regulation of lymphocyte function-associated antigen 1 adhesion and clustering as an alternate basis for coreceptor function. Proc. Natl. Acad. Sci. USA *102*, 12861–12866.

Schreiner, B., Mitsdoerffer, M., Kieseier, B.C., Chen, L., Hartung, H.P., Weller, M., and Wiendl, H. (2004). Interferon-beta enhances monocyte and dendritic cell expression of B7-H1 (PD-L1), a strong inhibitor of autologous T-cell activation: relevance for the immune modulatory effect in multiple sclerosis. J. Neuroimmunol. *155*, 172–182.

Sheppard, K.A., Fitz, L.J., Lee, J.M., Benander, C., George, J.A., Wooters, J., Qiu, Y., Jussif, J.M., Carter, L.L., Wood, C.R., and Chaudhary, D. (2004). PD-1 inhibits T-cell receptor induced phosphorylation of the ZAP70/CD3zeta signalosome and downstream signaling to PKCtheta. FEBS Lett. *574*, 37–41.

Shulman, Z., Gitlin, A.D., Targ, S., Jankovic, M., Pasqual, G., Nussenzweig, M.C., and Victora, G.D. (2013). T follicular helper cell dynamics in germinal centers. Science *341*, 673–677.

Suan, D., Nguyen, A., Moran, I., Bourne, K., Hermes, J.R., Arshi, M., Hampton, H.R., Tomura, M., Miwa, Y., Kelleher, A.D., et al. (2015). T follicular helper cells have distinct modes of migration and molecular signatures in naive and memory immune responses. Immunity *42*, 704–718.

Vinuesa, C.G., and Cyster, J.G. (2011). How T cells earn the follicular rite of passage. Immunity *35*, 671–680.

Vinuesa, C.G., Linterman, M.A., Yu, D., and MacLennan, I.C. (2016). Follicular helper T cells. Annu. Rev. Immunol. *34*, 335–368.

Wang, P., Shih, C.M., Qi, H., and Lan, Y.H. (2016). A stochastic model of the germinal center integrating local antigen competition, individualistic T-B interactions, and B cell receptor signaling. J. Immunol. *197*, 1169–1182.

Wang, Y., Shi, J., Yan, J., Xiao, Z., Hou, X., Lu, P., Hou, S., Mao, T., Liu, W., Ma, Y., et al. (2017). Germinal-center development of memory B cells driven by IL-9 from follicular helper T cells. Nat. Immunol. *18*, 921–930.

Weber, J.P., Fuhrmann, F., Feist, R.K., Lahmann, A., Al Baz, M.S., Gentz, L.J., Vu Van, D., Mages, H.W., Haftmann, C., Riedel, R., et al. (2015). ICOS maintains the T follicular helper cell phenotype by down-regulating Krüppel-like factor 2. J. Exp. Med. *212*, 217–233.

Wherry, E.J., and Kurachi, M. (2015). Molecular and cellular insights into T cell exhaustion. Nat. Rev. Immunol. *15*, 486–499.

Xiao, G., Deng, A., Liu, H., Ge, G., and Liu, X. (2012). Activator protein 1 suppresses antitumor T-cell function via the induction of programmed death 1. Proc. Natl. Acad. Sci. USA *109*, 15419–15424.

Xu, H., Li, X., Liu, D., Li, J., Zhang, X., Chen, X., Hou, S., Peng, L., Xu, C., Liu, W., et al. (2013). Follicular T-helper cell recruitment governed by bystander B cells and ICOS-driven motility. Nature *496*, 523–527.

Yokosuka, T., Takamatsu, M., Kobayashi-Imanishi, W., Hashimoto-Tane, A., Azuma, M., and Saito, T. (2012). Programmed cell death 1 forms negative costimulatory microclusters that directly inhibit T cell receptor signaling by recruiting phosphatase SHP2. J. Exp. Med. *209*, 1201–1217.

Yu, D., Rao, S., Tsai, L.M., Lee, S.K., He, Y., Sutcliffe, E.L., Srivastava, M., Linterman, M., Zheng, L., Simpson, N., et al. (2009). The transcriptional repressor Bcl-6 directs T follicular helper cell lineage commitment. Immunity *31*, 457–468.

Yusuf, I., Kageyama, R., Monticelli, L., Johnston, R.J., Ditoro, D., Hansen, K., Barnett, B., and Crotty, S. (2010). Germinal center T follicular helper cell IL-4 production is dependent on signaling lymphocytic activation molecule receptor (CD150). J. Immunol. *185*, 190–202.

Zinselmeyer, B.H., Heydari, S., Sacristán, C., Nayak, D., Cammer, M., Herz, J., Cheng, X., Davis, S.J., Dustin, M.L., and McGavern, D.B. (2013). PD-1 promotes immune exhaustion by inducing antiviral T cell motility paralysis. J. Exp. Med. *210*, 757–774.

STAR★METHODS

KEY RESOURCES TABLE

REAGENT or RESOURCE	SOURCE	IDENTIFIER
Antibodies		
CD185 (CXCR5) Biotin (Clone REA215)	Miltenyi Biotec	Cat#130-102-057; RRID: AB_2655798
PE/Cy7 anti-mouse CD279 (PD-1) Antibody (Clone RMP1-30)	BioLegend	Cat#109110; RRID: AB_572017
Ultra-LEAF Purified anti-human/mouse/rat CD278 (ICOS) antibody (Clone C398.4A)	BioLegend	Cat#313540; RRID: AB_2687114
PE anti-mouse CD274 (B7-H1, PD-L1) antibody (Clone 10F.9G2)	BioLegend	Cat# 124307; RRID: AB_2073557
BV421 Hamster Anti-Mouse CD183 Antibody (Clone CXCR3-173)	BD Biosciences	Cat#562937; RRID: AB_2687551
Bacterial and Virus Strains		
MSCV-UBC-GFP	Xu et al., 2013	N/A
PIB-IRES-RFP	This paper	N/A
PIB-IRES-EGFP	This paper	N/A
MSCV-LMP-AMT	Chen et al., 2015	N/A
Chemicals, Peptides, and Recombinant Proteins		
Recombinant Mouse PD-L1/B7-H1 Fc Chimera Protein, CF	R&D	Cat#Q9EP73
Albumin from chicken egg white	Sigma	Cat#5503
Lysozyme from hen egg white	Sigma	Cat#10837059001
NP-KLH (Keyhole Limpet Hemocyanin)	LGC Biosearch Technologies	Cat#N-5060
NP-OVAL (Ovalbumin)	LGC Biosearch Technologies	Cat#N-5051
NP-PE (Phycoerythrin)	LGC Biosearch Technologies	Cat#N-5070
NIP-BSA-Biotin	LGC Biosearch Technologies	Cat#N-1027
Critical Commercial Assays		
Fixation/Permeabilization Solution Kit	BD Biosciences	Cat#554714
Foxp3 / Transcription Factor Staining Buffer Set	eBioscience	Cat#00-5523-00
Ultra II DNA Library Prep Kit for Illumina	NEB	Cat#E7645S
Deposited Data		
Raw and analyzed data	This paper	GEO: GSE112066
Mouse reference genome NCBI build 37.2, MGSCv37	Genome Reference Consortium	https://www.ncbi.nlm.nih.gov/projects/genome/assembly/grc/mouse/
Experimental Models: Cell Lines		
Human: Platinum-E	Morita et al., 2000	N/A
Experimental Models: Organisms/Strains		
Mouse: *Pdcd1*$^{KI/KI}$: C57BL/6- *Pdcd1*$^{KI/KI}$	Xiao et al., 2012	N/A
Mouse: *Icos*$^{-/-}$: B6.129P2-*Icos*^{tm1Mak}/J	The Jackson Laboratory	Jax 4859
Mouse: *Icosl*$^{-/-}$: B6.129P2-*Icosl*^{tm1Mak}/J	The Jackson Laboratory	Jax 4657
Mouse: OT-II: B6.Cg-Tg(TcraTcrb)425Cbn/J	The Jackson Laboratory	Jax 4194
Mouse: MD4: C57BL/6-Tg(IghelMD4)4Ccg/J	The Jackson Laboratory	Jax 2595
Mouse: μMT: B6.129S2-*Ighm*^{tm1Cgn}/J	The Jackson Laboratory	Jax 2288
Mouse: CD45.1: B6.SJL-*Ptprc*^{a} *Pepc*^{b}/BoyJ	The Jackson Laboratory	Jax 002014
Mouse: *Sap*$^{-/-}$: B6.129S6-*Sh2d1a*^{tm1Pls}/J	Czar et al., 2001	Jax 025754
Mouse: dsRed: B6.Cg-Tg(CAG-DsRed*MST)1Nagy/J	The Jackson Laboratory	Jax 6051
Mouse: *Cd274*$^{-/-}$: C57BL/6- *Cd274*$^{-/-}$	This paper	N/A

(*Continued on next page*)

Continued		
REAGENT or RESOURCE	SOURCE	IDENTIFIER
Oligonucleotides		
VH186.2-1F: CTCTTCTTGGCAGCAACAGC	Liu et al., 2015	N/A
VH186.2-1R: GCTGCTCAGAGTGTAGAGGTC	Liu et al., 2015	N/A
VH186.2-2F: GTGTCCACTCCCAGGTCCAAC	Liu et al., 2015	N/A
VH186.2-2R: GTTCCAGGTCACTGTCACTG	Liu et al., 2015	N/A
CXCR3 shRNA-1: CTGCCTCAATCCGCTGCTCTAT	This paper	N/A
CXCR3 shRNA-2: AGCCGATGTTCTGCTGGTGTTA	This paper	N/A
Cd274 sgRNA: GTATGGCAGCAACGTCACGATGG	This paper	N/A
Cd274 genotyping-F: TAACAGGTGATCCGTTTCCTATG	This paper	N/A
Cd274 genotyping-F: CGTGATTCGCTTGTAGTCCG	This paper	N/A
Recombinant DNA		
MSCV-CXCR5-GFP	Xu et al., 2013	N/A
PIB-PD-1-EGFP	This paper	N/A
PIB-PD-1-RFP	This paper	N/A
PIB-PD-1(Y225F) -RFP	This paper	N/A
PIB-PD-1(Y248F) -RFP	This paper	N/A
PIB-ICOS-RFP	This paper	N/A
PIB-ICOS(Y181F) -RFP	This paper	N/A
Software and Algorithms		
TopHat2	Kim et al., 2013	http://ccb.jhu.edu/software/tophat
DESeq2	Love et al., 2014	https://bioconductor.org/packages/release/bioc/html/DESeq2.html

CONTACT FOR REAGENT AND RESOURCE SHARING

Further information and requests for resources and reagents should be directed to and will be fulfilled by the Lead Contact, Hai Qi (qihai@mail.tsinghua.edu.cn).

EXPERIMENT MODEL AND SUBJECT DETAILS

All mice were maintained under specific pathogen-free conditions and used in accordance of governmental and Tsinghua guidelines for animal welfare. Six- to 12-week-old, male and female, age and sex-matched mice were used for all experiments.

Platinum-E (Plat-E) Retroviral Packaging Cell Line was maintained in DMEM supplemented with 10% Fetal Bovine Serum, L-Glutamine, and penicillin/streptomycin. The cell line was female origin. Isolated $CD4^+$ T cells were activated *in vitro* by plate-bound anti-CD3 (8μg/ml) and anti-CD28 (8μg/ml) and after 2 days pipetted off and cultured in RPMI-1640 supplemented with 10% Fetal Bovine Serum, L-Glutamine, Sodium Pyruvate, penicillin/streptomycin, 2-Mercaptoethanol and 10 ng/ml IL-2. All cultures were incubated in a humidified chamber at 5% CO_2 and 37°C.

METHOD DETAILS

Animals and immunizations

B6 (Jax 664), CD45.1 (Jax 002014), μMT (Jax 2288), *Icos*$^{-/-}$ (Jax 4859), *Icosl*$^{-/-}$ (Jax 4657), dsRed-expressing (Jax 6051), T cell receptor transgenic OT-II mice (Jax 4194), and HEL-specific Ig-transgenic MD4 (Jax 2595) mice were from the Jackson Laboratory. *Pdcd1*$^{KI/KI}$ mice were as previously described (Xiao et al., 2012). *Sh2d1a*$^{-/-}$ (*Sap*$^{-/-}$) mice were a kind gift from Dr. Pamela Schwartzberg (Czar et al., 2001). Relevant mice were interbred to obtain *Pdcd1*$^{KI/KI}$-OT-II, *Pdcd1*$^{KI/KI}$-*Icos*$^{-/-}$, dsRed-expressing MD4 mice.

To examine T cell distribution patterns in GCs, 3-5 × 10^5 *in vitro* activated and retrovirally transduced OT-II T cells were intravenously transferred into B6 together with 5 × 10^5 naive dsred-MD4 B cells before subcutaneous immunization with 130 μg HEL-OVA (Sigma) protein plus 0.5 μg LPS (Sigma) in alum (Thermo Scientific). To examine polyclonal GC responses with OT-II T cells as the helper, 5 × 10^5 naive OT-II T cells were intravenously transferred into *Sap*$^{-/-}$ hosts followed by immunization with 50 μg NP-OVA (Biosearch Technologies) plus 1μg LPS in alum intraperitoneally. To measure GC responses in mixed BM chimeras, mice were intraperitoneally immunized with 100 μg NP-KLH (Biosearch Technologies) plus 1 μg LPS in alum.

Construction of bone marrow chimeras

B6 recipients were lethally irradiated by X-ray (5.5Gy × 2), and then intravenously transferred with a combination of 2-3 × 10^6 bone-marrow leukocytes from indicated donors mixed at indicated ratios. Chimeras were used for experiments after 8-week reconstitution.

Construction of *Cd274*$^{-/-}$ mice

For germline ablation of the *Cd274* gene in B6 mice, the CRISPR/Cas9 technique was used. Briefly, guide RNA targeting an appropriate site on exon 3 and close to the ATG start codon was co-injected together with Cas9-coding mRNA. PCR-positive F0 mice were screened by Sanger sequencing of the genomic region targeted by the guide RNA. A line that showed a 17-bp deletion resulting in a frameshift and a premature stop codon was chosen to further validate at the level of protein expression (Figure S5). This line was backcrossed with B6 mice for at least 3 generations before use in subsequent experiments. The sgRNA sequence is GTATGGCAG CAACGTCACGATGG. The genotyping primers used are: (primer-F) 5′-TAACAGGTGATCCGTTTCCTATG and (primer-R) 5′-CGTGATTCGCTTGTAGTCCG.

Retrovirus and *in vitro* transduction

Naive polyclonal T cells, OT-II T cells were isolated by CD4 Microbeads (Miltenyi Biotec) according to the manufacturer's protocols. $CD4^+$ T cells were activated *in vitro* by plate-bound anti-CD3 (8μg/ml) and anti-CD28 (8μg/ml). Target genes were cloned into MSCV or PIB-based, GFP- or RFP-tagged retroviral vectors. Retroviruses were packaged with the Plat-E system. For transduction, 1-2 × 10^6 T cells were typically spin-infected two times, one at 24 and the other at 48 h post activation, by 1,500 g centrifugation with desired viral supernatants containing 1 μg/ml polybrene (Sigma) and 10 ng/ml IL-2 (PeproTech) for 2 h at 32°C. Supernatants were then discarded and fresh media supplemented with10 ng/ml IL-2 were added. Transduced T cells were then cultured for another 3 days in complete media supplemented with 10 ng/ml IL-2 before sorting and transfer.

Immunoblotting

Previously activated or retrovirally transduced T cells were deprived of serum for 3 h. Approximately 2 × 10^6 T cells were suspended in serum-free RPMI-1640 medium, incubated with purified anti-ICOS (Biolegend) or CXCL13 (PeproTech) with or without PD-L1-Fc (R&D) of indicated concentration for 30 min in 37°C. Stimulated cells were quickly spun down and lysed by 2% SDS and loading buffer and then boiled at 100 C for 15 min. Proteins were separated by SDS-PAGE and transferred to PVDF membranes. Membranes were blocked with TBS containing 5% milk and 0.1% Tween-20. Antibodies for immunoblotting included rabbit anti–pAKT (S473), anti-AKT, anti-actin, anti-pSHP-2 (Y542), anti-SHP2 (Cell Signaling Technology). Appropriate HRP-conjugated secondary reagents were from Jackson Immunoresearch Laboratories.

Chemokine-stimulated T cell polarization assay

Activated T cells were retrovirally transduced with MSCV-CXCR5-GFP and PIB-PD-1-RFP. GFP^+RFP^+ co-transduced cells were sorted to > 90% purity. After resting in complete RPMI-1640 media for 2-3 hours, cells were re-suspended to a density of 3 × 10^6 cells/ml in the same media supplemented with or without 1 μg/ml recombinant CXCL13 (Peprotech) dropped onto L-Lysine-coated slides that were pre-incubated with PD-L1-hIgGFc or control human IgG. After incubation at 37°C for 30 min, cells were fixed with 1% paraformaldehyde and stained with AF568 Phalloidin (Invitrogen) to visualize F-actin. Slides were mounted with the ProlongGold Antifade reagent (Invitrogen) and examined with an Olympus FV1000 upright microscope using × 20 air immersion lens. Cells with an elongated shape (ratio of the cell length and width > 1.5) are considered as polarized.

Follicular homing assay and immunohistochemistry

Activated T cells were retrovirally transduced with MSCV-CXCR5-GFP alone or in combination with PIB-target gene-RFP. GFP^+ or GFP^+RFP^+ cells were sorted to > 90% purity and intravenously injected into B6 or desired hosts (2-3 × 10^6 cells per mouse). 24 h later, splenic tissue blocks were fixed with 1% paraformaldehyde. Immunohistochemical staining was conducted to examine T cell distribution patterns according to protocols previously described (Xu et al., 2013). Staining reagents included EF450 anti-CD3, EF660 anti-CD3, EF450 anti-IgD, AF647 anti-IgD (eBiosience), anti-GFP (ab6556, Abcam), AF488 donkey-anti-rabbit IgG (Invitrogen). Slides were mounted with the ProlongGold Antifade reagent (Invitrogen) and examined with an Olympus FV1000 upright microscope using × 20 air immersion lens. Care was to taken to exclude consecutive sections from the same lymph node or spleen to avoid under-sampling, and multiple animals from multiple independent experiments were always used for quantitating follicular homing coefficients. The representative images were pseudo-colored and output from Imaris (Bitplane).

Flow cytometry

To phenotype distinct cell populations by flow cytometry, draining lymph node cells or splenocytes were washed with PBS, blocked with 20cμgcml^{-1} 2.4G2 (BioXcell), and then stained with indicated antibodies in MACS buffer (PBS supplemented with 1% FBS and 5cmM EDTA). Staining reagents included AF700 anti-CD4, APC-Cy7 anti-CD19, APC-Cy7 anti-B220, PerCP-Cy5.5 anti-IgD, APC anti-IgD, PE-Cy7 anti-CD95, BV510 anti-CD95, BV510 anti-CD138, AF647 anti-BCL6, APC-Cy7 anti-CD45.2, APC anti-CD45.2, PerCP-Cy5.5 anti-CD45.1, FITC anti-CD45.1, BV421 and-CXCR3, streptavidin-BV421 and PE anti-IgM^a from BD Biosciences; FITC anti-GL7, EF450 anti-GL7, AF700 anti-B220, FITC anti-IgD, streptavidin-PerCP-Cy5.5, streptavidin-PE, streptavidin-APC, PE anti-IL-21 and APC anti-IL-21 from eBioscience; APC anti-PD1, PE-Cy7 anti-PD1, biotinylated anti-ICOS, PE anti-ICOS and

PE anti-CD40L from Biolegend; biotinylated anti-CXCR5 and PE anti-CXCR5 from Miltenyi. Isotype-matched control antibodies were purchased from these respective vendors. Surface staining was done on ice with primary reagents incubated for 30cminutes (90 minutes for CXCR5) followed by secondary reagents for 30cminutes with washes in-between. For intracellular staining of IL-21 or Bcl-6, the CytoFix/Perm kit (BD Biosciences) or the Foxp3/Transcription Factor Staining Buffer Set (eBioscience) was respectively used according to manufacturers' protocols. To detect NP-binding cells, $NP_{(38)}$- or $NP_{(44)}$-PE were used. All cytometric data were collected on an LSR II or Aria III cytometer (BD Biosciences) and analyzed with the FlowJo software (TreeStar). Dead cells and non-singlet events were excluded from analyses based on staining of 7-amino-actinomycin D (7-AAD) (Biotium) or Zombie Yellow (Biolegend) and characteristics of forward- and side-scattering.

V_H186.2 sequence analysis of NP-specific GC B cells

To analyze V_H sequences of NP-specific GC B cell subsets, the antigen-binding cells pooled from 3-4 mice per group each experiment were identified by staining with biotinylated $NIP_{(15)}$-BSA (Biosearch Technologies) at 330 ng/ml for 1 hours on ice and then with streptavidin APC. NIP-binding$^+$GL7$^+$FAS$^+$CD19$^+$ GC B cells were sorted 13 days after intraperitoneal NP-KLH immunization. After incubation at 60°C for 5 min in the lysis buffer, lysates of multiple 100-cell sorts per experiment was subjected to reverse-transcription by with the Superscript cDNA Synthesis Kit (Invitrogen) using the manufacturer's suggested protocol. V_H186.2 fragments were amplified from the complementary DNA by nested PCR using the following primers (first sense: 5′-CTCTTCTTGGCAGCAACAGC, first antisense: 5′-GCTGCTCAGAGTGTAGAGGTC; second sense: 5′-GTGTCCACTCCCAGGTCCAAC, second antisense: 5′-GTTCCAGGTCACTGTCACTG). PCR products (400 base pairs) were purified by gel electrophoresis and cloned into a T vector (Takara), and individual bacterial colonies were picked for sequencing. Identical V_H sequences were counted only once as one clone, and the final result was compiled with unique clones for each category.

CXCR3 knockdown

The mir-30 microRNA-based, Ametrine-tagged, shRNA-expressing MSCV-LMP vector was a kind gift from Dr. Yun-Cai Liu. Five CXCR3-targeting shRNA sequences were initially obtained from the CSHL website (http://katahdin.cshl.org/homepage/siRNA/RNAi.cgi?type=shRNA) and tested for the knockdown efficiency by surface staining of CD4$^+$ T cells activated and transduced *in vitro*. Two highly efficient ones were used in subsequent experiments: (shRNA-1) CTGCCTCAATCCGCTGCTCTAT and (shRNA-2) AGCCGATGTTCTGCTGGTGTTA.

RNA-seq

To conduct transcriptomic RNA-seq analyses on Tfh cells, we modified the protocol initially developed for single-cell RNA-seq to accommodate 200 sorted cells as previously described (Wang et al., 2017). cDNAs were sheared by Covaris and subjected to Illumina TruSeq sequencing library preparation. All libraries were sequenced on a HiSeq 2500 sequencer (Illumina) in the SE-50 mode by Tsinghua core facility.

QUANTIFICATION AND STATISTICAL ANAYSIS

For all relevant animal experiments, age- and sex-matched mice were randomly chosen to be in different treatment groups. Each group typically contained 3 to 5 animals, and 2 to 6 independent experiments were done for each assay. No blinding was involved in this work. Unless indicated otherwise, two-tailed t tests in Prism software (Graphpad) were used to compare endpoint means of different groups. Differences between groups were considered significant for p values < 0.05.

For RNA-seq data analysis, after raw data were processed with CASAVA (Illumina) to generate fastq files, sequence reads were aligned to the *Mus musculus* reference genome using TopHat2. Taking into account only genes that have an average number of reads of at least 1, differential expression between any two cell subsets was calculated by the DESeq2 software (Bioconductor) with a call threshold set at $P_{adj}<0.01$. Two independent immunization experiments were conducted, and in each experiment spleens of 3 immunized mice were pooled to obtain three independent sorts of 200 cells for each Tfh genotype. As a result, for each Tfh genotype a total of 6 independent replicates were analyzed.

DATA AND SOFTWARE AVAILABILITY

RNA-seq data are deposited in GEO database under ID code GSE112066.

Immunity, Volume *49*

Supplemental Information

PD-1 Controls Follicular T Helper Cell Positioning and Function

Jingwen Shi, Shiyue Hou, Qian Fang, Xin Liu, Xiaolong Liu, and Hai Qi

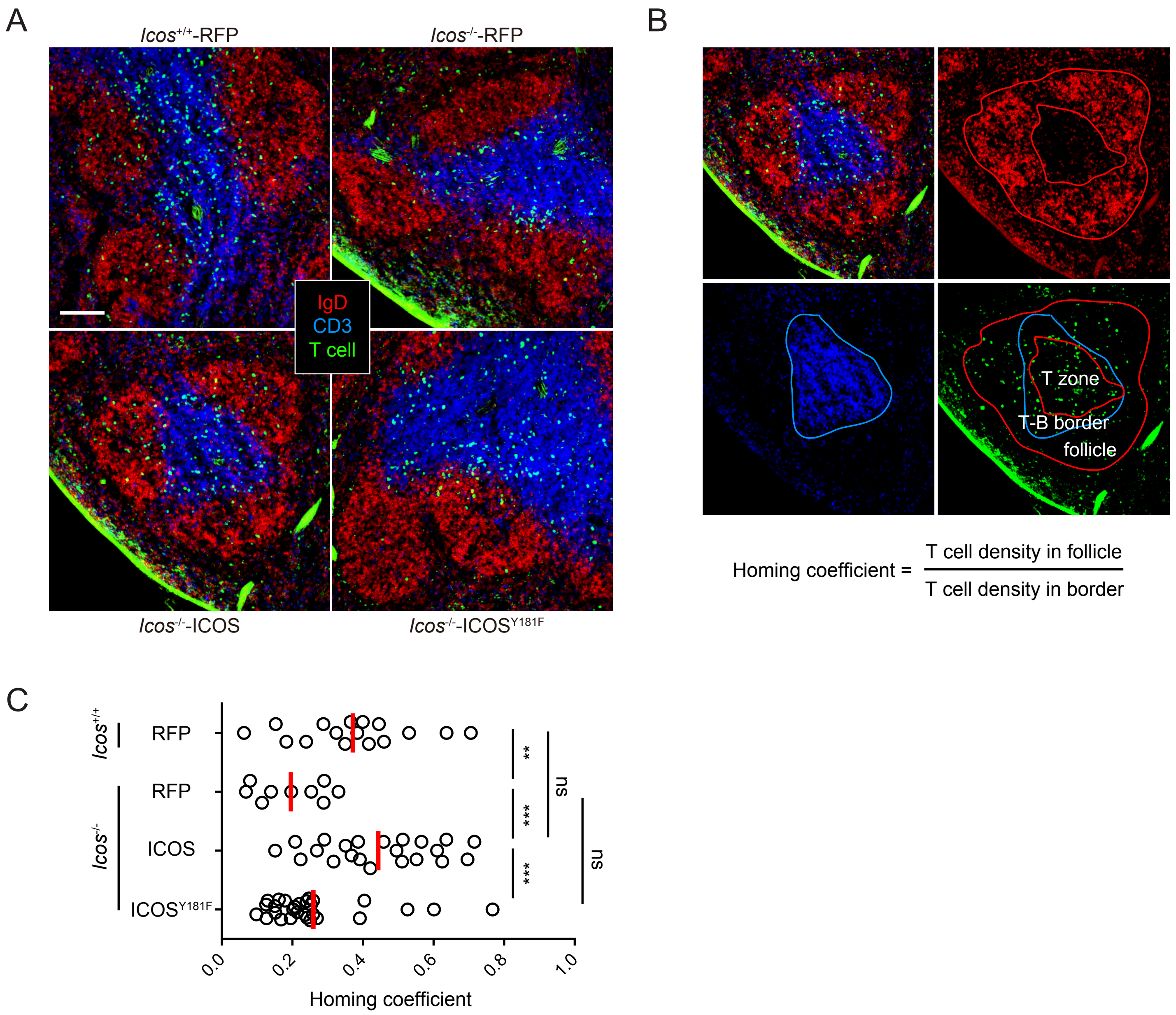

Figure S1. ICOS-driven follicular recruitment requires its PI3K-activating Y181-based motif. Related to Figure 1.

T cells of indicated genotypes were retrovirally co-transduced with a vector co-expressing CXCR5 and GFP and another vector expressing empty RFP, wildtype ICOS or mutant ICOS^{Y181F}. These cells were transferred into B6 mice (2-3×10^{6} sorted GFP^{+}RFP^{+} cells per mouse).

(A) GFP^{+} T-cell distribution patterns in the spleen 24h after transfer. Scale bar, 100 µm.

(B) Derivation of the follicular homing coefficient parameter. The outer boundary of the T-cell zone is defined as endogenous CD3^{+} cells farthest into the follicle (blue line), while the inner boundary of the follicle is defined as endogenous IgD^{+} cells farthest into the T-cell zone (inner red line). The T–B border area is thus the region in-between endogenous CD3^{+} cells farthest into the follicle and endogenous IgD^{+} cells farthest into the T-cell zone.

(C) Homing coefficients of the four groups in (A). Each symbol represents one section. Data represent four independent experiments, each involving at least 3 recipients.

** P<0.01; *** P<0.0001; ns, not significant.

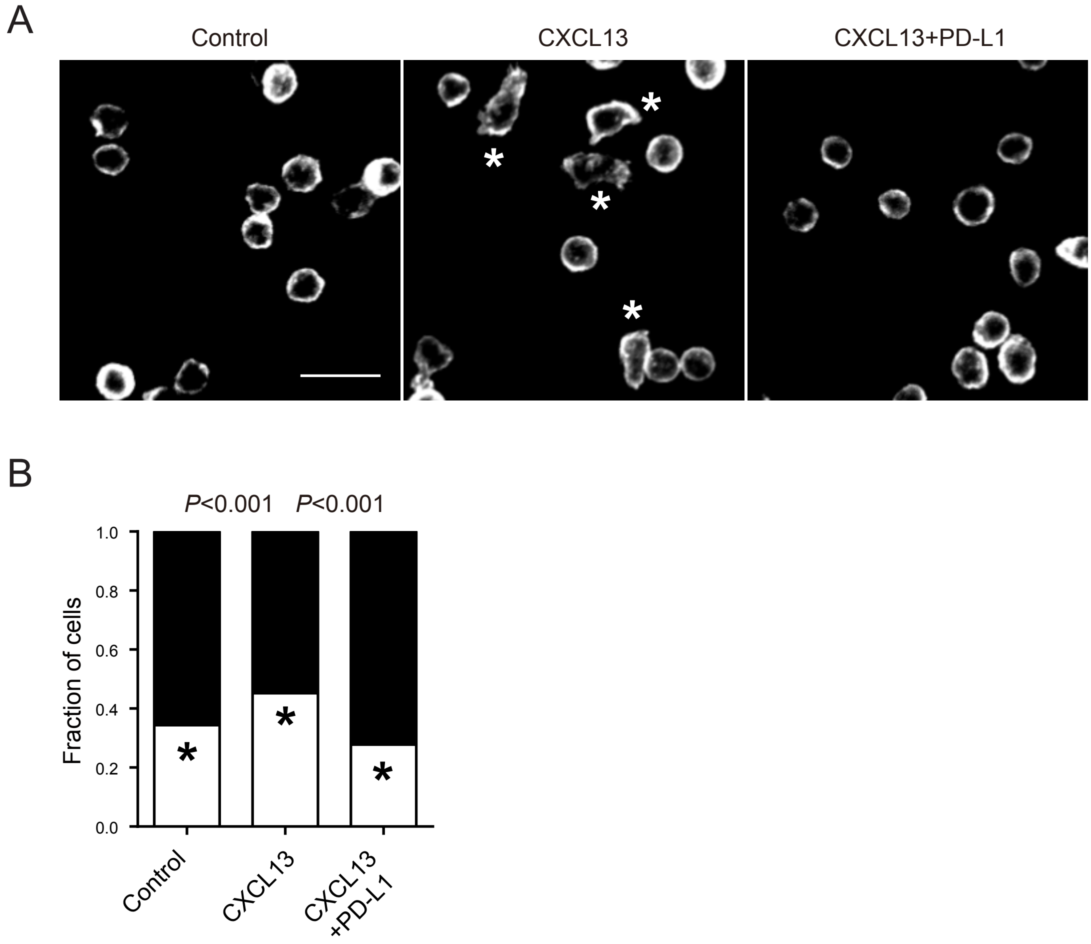

Figure S2. PD-1 inhibits CXCL13-stimulated T-cell polarization. Related to Figure 1.

(A) Typical morphologies revealed by Phalloidin staining of F-actin in co-transduced CXCR5^{+}PD-1^{+} T cells that were not stimulated (Control), CXCL13-stimulated (CXCL13), or CXCL13-stimulated in the presence of PD-L1-Fc (CXCL13+PD-L1). Asterisks mark polarized cells. Scale bar, 20 µm.

(B) Fractions of polarized (*) and non-polarized cells among the 3 groups, with a total of 1629 (Control), 1375 (CXCL13), and 1815 (CXCL13+PD-L1) cells enumerated in 4 independent experiments. *P* values from Fisher's exact tests.

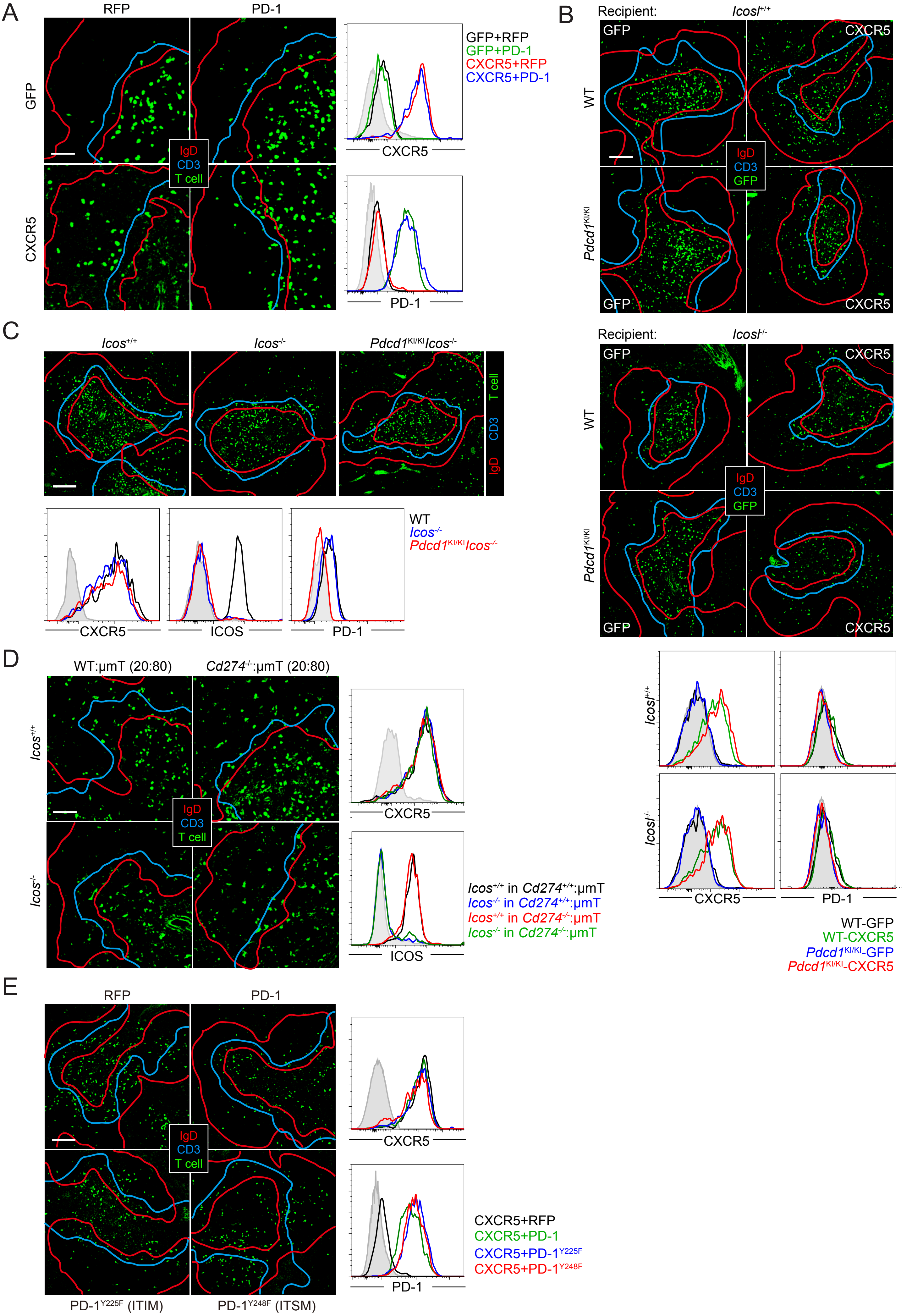

A
RFP
PD-1
GFP
CXCR5
IgD
CD3
T cell
GFP+RFP
GFP+PD-1
CXCR5+RFP
CXCR5+PD-1
CXCR5
PD-1
B
Recipient:
Icosl+/+
WT
Pdcd1KI/KI
GFP
CXCR5
IgD
CD3
GFP
Recipient:
Icosl-/-
Icosl+/+
Icosl-/-
CXCR5
PD-1
WT-GFP
WT-CXCR5
Pdcd1KI/KI-GFP
Pdcd1KI/KI-CXCR5
C
Icos+/+
Icos-/-
Pdcd1KI/KIIcos-/-
T cell
CD3
IgD
WT
Icos-/-
Pdcd1KI/KIIcos-/-
CXCR5
ICOS
PD-1
D
WT:μmT (20:80)
Cd274-/-:μmT (20:80)
Icos+/+
Icos-/-
IgD
CD3
T cell
CXCR5
ICOS
Icos+/+ in Cd274+/+:μmT
Icos-/- in Cd274+/+:μmT
Icos+/+ in Cd274-/-:μmT
Icos-/- in Cd274-/-:μmT
E
RFP
PD-1
IgD
CD3
T cell
PD-1Y225F (ITIM)
PD-1Y248F (ITSM)
CXCR5
PD-1
CXCR5+RFP
CXCR5+PD-1
CXCR5+PD-1Y225F
CXCR5+PD-1Y248F

Figure S3. Definition of T-zone and follicular borders for quantitative homing analyses and surface levels of CXCR5, PD-1 and ICOS on transduced cells. Related to Figure 1.
The boundaries of T-cell zones, follicles and T-B border areas are defined as in Figure S1B. Outlines of T-cell zones, follicles, and T-B border areas for representative images from all the homing assays shown in Figures 1D (**A**), 2A (**B**), 2C (**C**), 3A (**D**) and 4A (**E**), respectively. Surface expression of CXCR5 and PD-1, together with ICOS in certain experiments, are also shown for the transferred cells in corresponding experiments.

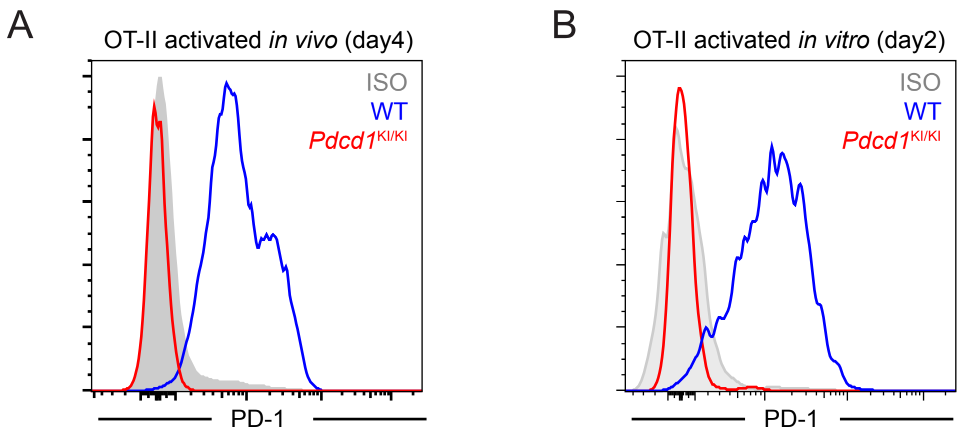

Figure S4. Disabled PD-1 upregulation in *Pdcd1*$^{KI/KI}$ T cells. Related to Figure 2.

Surface PD-1 expression by wildtype or *Pdcd1*$^{KI/KI}$ OT-II T cells in adoptive B6 hosts 4 d after ovalbulmin immunization (A), or 2 d after in vitro activation with plate-bounded anti-CD3 and anti-CD28 (B). Grey histograms, isotype control. Data represent three independent experiments.

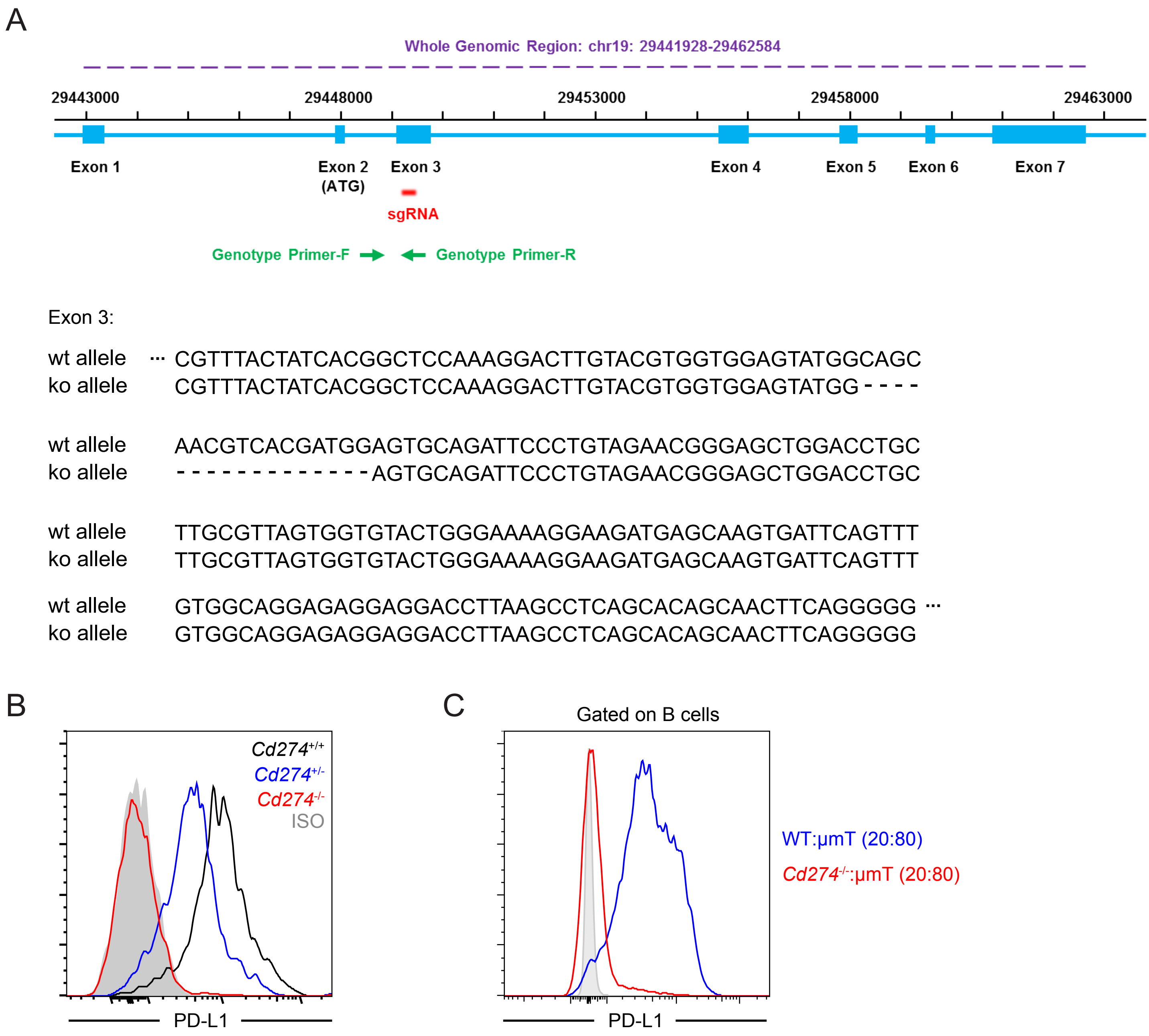

Figure S5. Genetic ablation of *Cd274*. Related to Figure 3.

(A) Schematic presentation of CRISPR/Cas9-mediated targeting and genotyping strategy for genomic deletion of *Cd274* (top) and sequence alignment of the wildtype allele and the knockout allele used in this study (bottom). Nucleotide coordinates according to NCBI and exon distribution of the *Cd274* locus are shown. The exon 3 was targeted by an sgRNA (red), and 2 green arrows mark the positions of the genotyping primers (see Methods for primer sequences).

(B) PD-L1 expression by naïve B cells from mice of the indicated genotypes.

(C) PD-L1 expression by B cells from mixed BM chimeras of indicated types, as used in Figure 3. Grey histogram, isotype control.

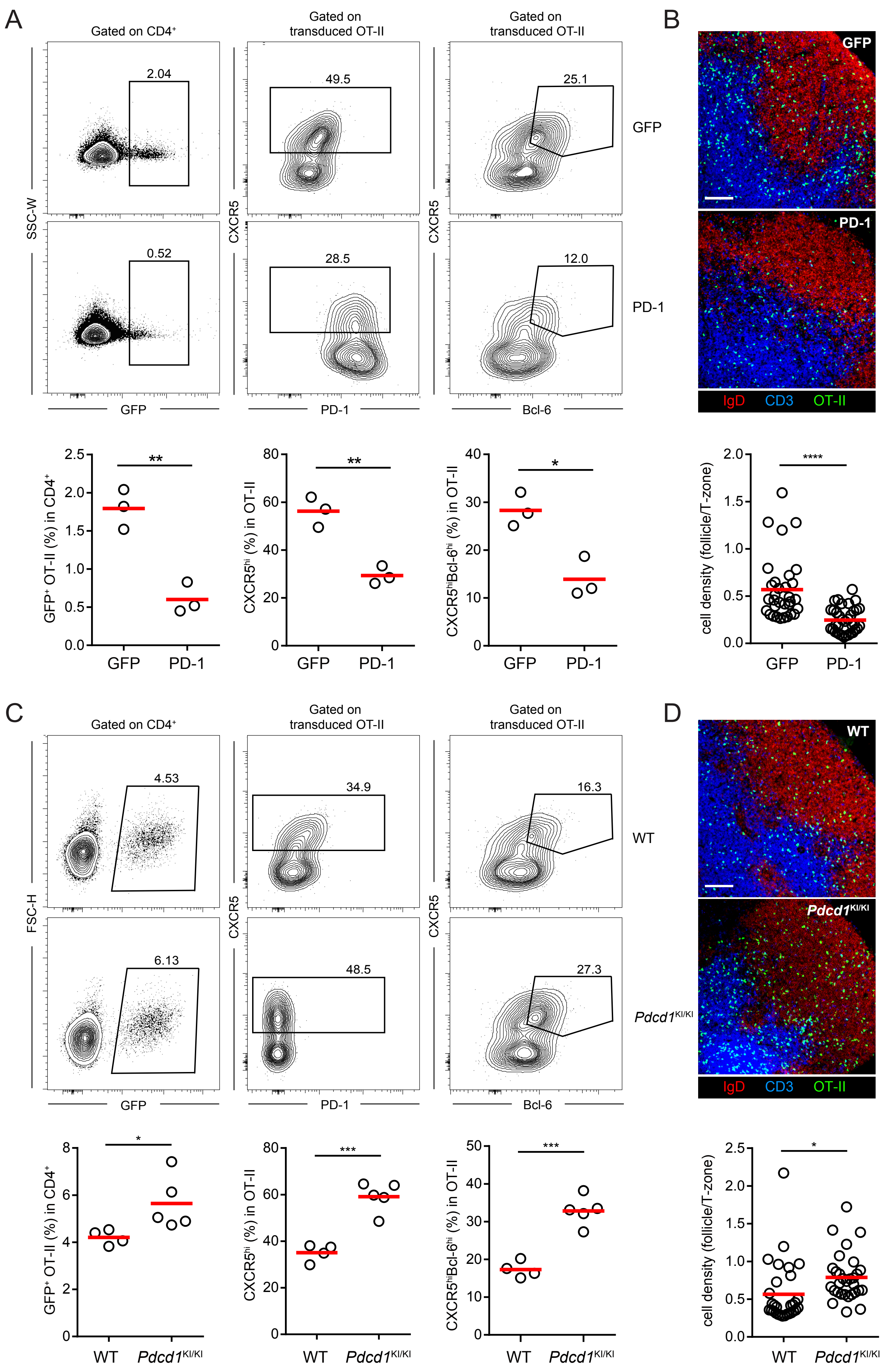
A
Gated on CD4+
Gated on transduced OT-II
Gated on transduced OT-II
2.04
49.5
25.1
GFP
0.52
28.5
12.0
PD-1
SSC-W
CXCR5
CXCR5
GFP
PD-1
Bcl-6
B
GFP
PD-1
IgD
CD3
OT-II
GFP+ OT-II (%) in CD4+
CXCR5hi (%) in OT-II
CXCR5hiBcl-6hi (%) in OT-II
cell density (follicle/T-zone)
**
**
*
****
GFP
PD-1
C
Gated on CD4+
Gated on transduced OT-II
Gated on transduced OT-II
4.53
34.9
16.3
WT
6.13
48.5
27.3
Pdcd1KI/KI
FSC-H
CXCR5
CXCR5
GFP
PD-1
Bcl-6
D
WT
Pdcd1KI/KI
IgD
CD3
OT-II
GFP+ OT-II (%) in CD4+
CXCR5hi (%) in OT-II
CXCR5hiBcl-6hi (%) in OT-II
cell density (follicle/T-zone)
*
***
***
*
WT
Pdcd1KI/KI

Figure S6. PD-1 inhibits Tfh development. Related to Figure 5.

(A) OT-II T cells transduced with a vector expressing PD-1 or control GFP were transferred into B6 mice (5×10^5 sorted GFP^+ cells per mouse), and then these mice were subcutaneously immunized with OVA protein. Expansion of GFP^+ OT-II cells, measured by their frequencies in total CD4 T cells, and Tfh frequencies, as measured by $CXCR5^{hi}$ or $CXCR5^{hi}Bcl\text{-}6^{hi}$ fractions in OT-II cells were assayed in the draining lymph node 4 d post immunization.

(B) OT-II distribution patterns and density ratios between the follicle and the T-zone in draining lymph nodes in (A).

(C) GFP-transduced wildtype or *Pdcd1*$^{KI/KI}$ OT-II T cells were transferred into B6 mice (5×10^5 sorted GFP^+ cells per mouse), and the recipients were subcutaneously immunized with OVA protein. Expansion of GFP^+ OT-II cells, measured by their frequencies in total CD4 T cells, and Tfh frequencies, as measured by $CXCR5^{hi}$ or $CXCR5^{hi}Bcl\text{-}6^{hi}$ fractions in OT-II cells were assayed in the draining lymph node 4 d post immunization.

(D) OT-II distribution patterns and density ratios between the follicle and the T-zone in draining lymph nodes in (C).

Scale bar, 100 μm. Flow cytometry data are from one of 3 independent experiments with similar results (3-4 recipient mice per condition per experiment). Data on cell density ratios are pooled from 3 independent experiments. * $P<0.05$; ** $P<0.01$; **** $P<0.0001$.

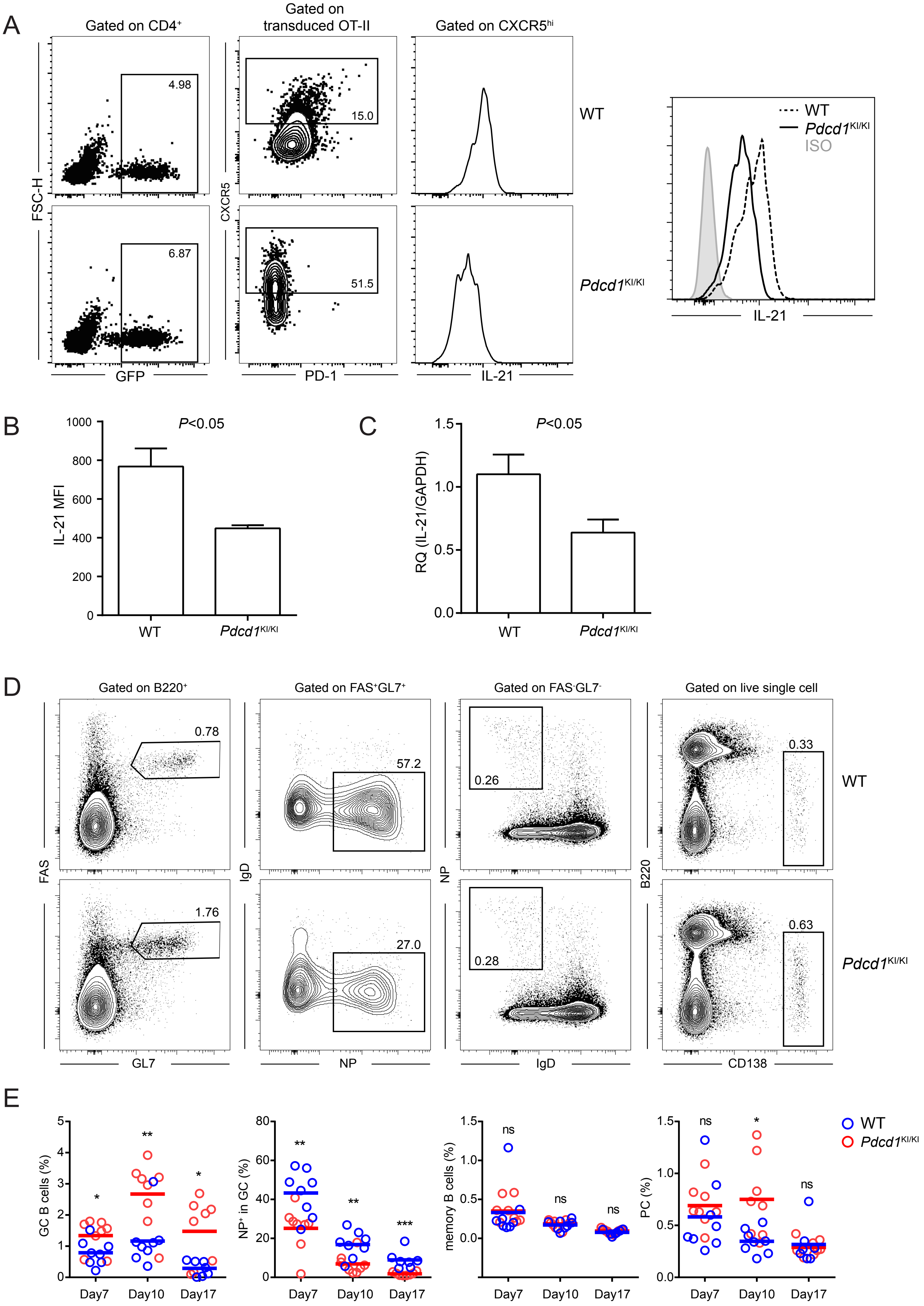

A
Gated on CD4⁺
Gated on transduced OT-II
Gated on CXCR5hi
4.98
6.87
15.0
51.5
WT
Pdcd1KI/KI
ISO
FSC-H
GFP
CXCR5
PD-1
IL-21
B
IL-21 MFI
P<0.05
WT
Pdcd1KI/KI
C
RQ (IL-21/GAPDH)
P<0.05
D
Gated on B220⁺
Gated on FAS⁺GL7⁺
Gated on FAS⁻GL7⁻
Gated on live single cell
0.78
1.76
57.2
27.0
0.26
0.28
0.33
0.63
FAS
GL7
IgD
NP
B220
CD138
E
GC B cells (%)
NP⁺ in GC (%)
memory B cells (%)
PC (%)
Day7
Day10
Day17
ns

Figure S7. *Pdcd1*$^{KI/KI}$ Tfh cells are impaired in IL-21 production and causes abnormal GC responses. Related to Figure 7.

(A) GFP-transduced wildtype or *Pdcd1*$^{KI/KI}$ OT-II T cells were transferred into B6 mice (5×10^5 sorted GFP$^+$ cells per mouse) together with 5×10^5 dsRed-expressing MD4 B cells, and the recipients were subcutaneously immunized with HEL-OVA. Strategies for gating GFP$^+$CXCR5^{hi} OT-II Tfh cells and intracellular IL-21 staining of Tfh cells of the two genotypes are shown.

(B) Mean fluorescence intensity (MFI, mean±sem) of IL-21 staining in Tfh cells of indicated genotypes (n=3). One of two experiments with similar results is shown.

(C) Relative IL-21 expression in wildtype or *Pdcd1*$^{KI/KI}$ OT-II Tfh cells as detected by quantitative RT-PCR. Data are mean ± s.e.m of 5 independent experiments.

(D) *Sap*$^{-/-}$ mice received 5×10^5 wildtype or *Pdcd1*$^{KI/KI}$ OT-II T cells and were intraperitoneally immunized with NP-OVA. From left to right, gating of GC B cells, NP-binding cells in GCs, memory B cells, and plasma cells, as assayed 7 d post immunization.

(E) Frequencies of GC B cells in total B220$^+$ cells, NP-specific cells in total GC B cells, NP-specific memory B cells in non-GC B cells, and plasma cells in total splenocytes 7, 10, and 17 d after immunization. Each symbol denotes one mouse. Data are pooled from 3 independent experiments.

* $P<0.05$, ** $P<0.01$, *** $P<0.001$.
